# The *N*‐Glycome to Differentiate Mesenchymal Stem Cells Upon Chondrogenic Differentiation, Dedifferentiation, and Senescence

**DOI:** 10.1002/pmic.70124

**Published:** 2026-03-25

**Authors:** Houda Montacir, Karina Biskup, Michael Sittinger, Rudolf Tauber, Véronique Blanchard

**Affiliations:** ^1^ Corporate Member of Freie Universität Berlin Humboldt‐Universität zu Berlin Berlin Institute of Health Institute of Laboratory Medicine Clinical Chemistry and Pathobiochemistry Charité – Universitätsmedizin Berlin Berlin Germany; ^2^ Department of Biology, Chemistry and Pharmacy Freie Universität Berlin Berlin Germany; ^3^ Medical School Berlin Department of Human Medicine Berlin Germany; ^4^ Corporate member of Freie Universität Berlin Humboldt‐Universität zu Berlin Berlin Institute of Health Tissue Engineering Laboratory & Berlin‐Brandenburg Center for Regenerative Therapies Dept. of Rheumatology and Clinical Immunology Charité – Universitätsmedizin Berlin Berlin Germany

**Keywords:** aging, chondrogenic differentiation, dedifferentiation, glycomics, MALDI‐TOF, mesenchymal stem cells, *N*‐glycans, permethylation

## Abstract

Mesenchymal stem cells (MSCs) are adult stem cells able to self‐renewal or differentiation into different cell types, including chondrocytes. *N‐*Glycans are post‐translational modifications of glycoproteins that contribute to vital cell functions. In this work, we examined the cell surface *N*‐glycome of human MSCs isolated from bone marrow to identify biomarkers for chondrogenic differentiation. In addition, we investigated for the first time the *N*‐glycome of MSCs upon dedifferentiation and aging. Cell surface glycoproteins were released by tryptic digestion, then *N*‐glycans were enzymatically cleaved, purified, permethylated, and analyzed by MALDI‐TOF‐MS combined with exoglycosidase digestions. We were able to detect 68 signals, comprising paucimannose, high‐mannose, hybrid, and complex‐type *N*‐glycans, as well as structures containing polylactosamine motifs. A statistically significant decrease in antennarity and galactosylation, accompanied by an increase in sialylation, was observed during chondrogenic differentiation. Comparison of MSCs at passage 3 and passage 8 revealed increased levels of paucimannosylation, hybrid‐type glycans, and sialylation, together with decreased biantennarity, bigalactosylation, and core‐fucosylated *N*‐glycans. Dedifferentiated MSCs exhibited a stem cell‐like *N*‐glycosylation profile, although statistically significant differences were still detected. These data show that the *N*‐glycosylation profile of MSCs may serve as an indicator of the differentiation stage, dedifferentiation, and aging for the quality control of MSCs.

**Abbreviations**
bFGF: basic‐fibroblast growth factor
CD: cluster of differentiation
CE‐LIF: capillary electrophoresis coupled with laser‐induced fluorescence
COL1A1: collagen type 1 A1
ECM: extracellular matrix
ESCs: embryonic stem cells
F: fucose
GAPDH: glyceraldehyde‐3‐phosphate dehydrogenase
Glc: glucose
GlcNAc: *N*‐acetyl glucosamine
H: hexose
LacNAc: *N*‐acetyl lactosamine
MAA: *Maackia amurensis*
MALDI‐TOF: Matrix‐Assisted Laser Desorption/Ionization Time‐of‐Flight
MSCs: mesenchymal stem cells
N: *N*‐acetyl hexosamine
S: *N*‐acetyl neuraminic acid
SD: standard deviation
SNA: *S. nigra* agglutinin
SOX9: sex‐determining region Y‐box‐9
SSEA: stage‐specific embryonic antigen
ST6GAL1: β‐galactoside a2,6‐sialyltransferase 1
TGF‐β3: transforming growth factor‐β3

## 1 Introduction

Bone marrow MSCs are non‐hematopoietic adult stem cells capable of self‐renewal and differentiation into a variety of cell types, including adipocytes, osteocytes, and chondrocytes. The minimal criteria defining MSCs include adherent growth in standard culture conditions, ability to differentiate into various progenies, the presence of CD105, CD73, and CD90, and the lack of expression of several specific cell surface markers [1]. MSCs are easy to isolate, culture, and expand both in vitro and in vivo [2]. They are genetically stable and poorly immunogenic, as they do not produce HLA‐class II or costimulatory molecules [2]. Accordingly, MSCs are considered highly promising candidates for the treatment of a wide spectrum of diseases. Clinically, MSCs are used for their immunomodulatory properties and as a tool to repair diseased tissues. The therapeutic safety of autologous and allogeneic MSCs and their differentiated progenies has been explored in clinical trials for the treatment of numerous conditions, including rheumatoid arthritis [3], traumatic spinal cord injury [4], Crohn´s disease [5], respiratory failure from COVID‐19 [6], and Alzheimer´s disease [7].

Cell surface antigens are used as biomarkers to verify the quality and differentiation stage of MSCs during manufacturing prior to therapeutic use. However, most well‐characterized stem cell surface markers, such as CD44, CD73, CD90, CD105, and CD166, are not specific to MSCs [1]. Other markers such as Stro‐1, stage‐specific embryonic antigen (SSEA)‐1, SSEA‐4, CD271, and CD146 may exhibit a fluctuating expression depending on the source of the MSCs [1]. The same applies to the transcriptome, which may vary according to the MSC source [8]. Hence, superior biomarkers are needed for quality control of MSCs and their progenies.

**Significance of the Study**

*N‐*Glycosylation is a post‐translational protein modification that is modulated in the course of development as well as in health and disease. Cell surface *N*‐glycosylation actively contributes to cellular functions, including cell–cell communication and signal transduction. Biomarkers currently used to check the quality of stem cells prior to patient treatment lack specificity. To address the need for improved stem cell biomarkers, we characterized the cell surface *N*‐glycome of human MSCs isolated from bone marrow to identify biomarkers for chondrogenic differentiation. In addition, we investigated for the first time the *N*‐glycome of MSCs upon dedifferentiation and aging. From the differences observed between the different MSC preparations (undifferentiated, chondrogenic differentiated, dedifferentiated, and late passage), we concluded that the cell surface *N*‐glycome could be used as an indicator of the differentiation stage for the quality control of MSCs.

*N*‐Glycosylation is the most prevalent post‐translational modification of proteins. It occurs at asparagine residues of newly synthesized polypeptides following the consensus sequence asparagine‐X‐serine/threonine, where X may be any amino acid except proline. *N*‐Glycosylation of cell surface glycoproteins is actively involved in essential cellular functions such as cell–cell communication, signal transduction, and development [9]. For example, in embryonic stem cells (ESCs), glycans contribute to major signaling pathways; specifically, the glycan motif GalNAcβ1‐4GlcNAc maintains the naïve state through LIF/STAT signaling [10]. In addition, β‐galactoside α2,6‐sialyltransferase 1 (ST6GAL1) and α2,6 sialylated glycans help retain ESC stemness by regulating OCT3/4 and SOX2 [11]. We previously studied the *N*‐glycan signatures of MSCs during adipogenic differentiation and reported an increase in sialylation and biantennary fucosylated structures [12]. Moreover, we demonstrated that hepatogenic differentiation of ESCs is accompanied by an increase in complex‐type *N*‐glycosylation, particularly in fully galactosylated structures [13]. In the present work, cell surface *N*‐glycosylation of MSCs was analyzed by comparing freshly isolated MSCs, MSCs after chondrogenic differentiation, chondrogenic dedifferentiated MSCs, and MSCs at passages 3 and 8 to reflect cell aging. To our knowledge, this is the first study of bone marrow MSC senescence and dedifferentiation from a glycomics perspective.

## 2 Materials and Methods

Reagents were purchased from Sigma–Aldrich (Taufkirchen, Germany) unless stated otherwise.

### 2.1 Ethics Statement and MSCs Isolation

The study was approved by the institutional ethical committee of the Charité‐Universitätsmedizin Berlin (EA1/131/07). In accordance with the requirements of the ethical review board, written informed consent was obtained from all participants. Human MSCs were isolated from bone marrow aspirates as described previously [14, 15] (see Supporting Information).

### 2.2 Release of Cell Surface Glycopeptides and *N*‐glycan Cleavage

Cell surface glycopeptides were cleaved as reported in Hamouda et al. [16]. Briefly, approximately 4 × 10^6^ cells were harvested and washed four times with PBS (15 min at 600 rpm). The cell pellets were then resuspended in 500 µL of cold PBS containing trypsin (2.5 mg/mL). Cell surface glycopeptides were released by incubation at 150 rpm for 15 min at 37°C and subsequently separated from the pellet by centrifugation at 15,000 rpm for 15 min at 4°C. Samples were then incubated at 99°C for 5 min to inactivate the trypsin. *N*‐Glycans were cleaved from the glycopeptides overnight at 37°C using 0.5 U PNGase F (Roche, Basel, Switzerland). Finally, the glycans were isolated from the de‐*N*‐glycosylated peptides using C18 cartridges (Alltech, Deerfield, IL), desalted on a graphitized carbon column (Alltech, Deerfield, IL), and dried in a vacuum centrifuge.

### 2.3 Exoglycosidase Digestions

*N*‐Glycans were dissolved in 100 mM sodium acetate (pH 5.0). They were subjected to the following sequential exoglycosidase (Prozyme, CA) digestions in the following order: 3 U/mL *A. ureafaciens* neuraminidase, 0.8 U/mL β(1‐4) galactosidase from *S. pneumoniae*, 5.4 mU/mL almond meal α(1‐,3,4) fucosidase, another 0.8 U/mL β(1‐4) galactosidase, 0.75 U/mL bovine kidney α(1‐2,3,4,6) fucosidase, and α‐mannosidase from *Canavalia ensiformis*. After heat inactivation at 95°C for 5 min, samples were permethylated and lyophilized.

### 2.4 Permethylation and Mass Spectrometry

*N*‐Glycans were permethylated using a freshly prepared NaOH/DMSO mixture and iodomethane as the methylating agent [17, 18]. The reaction was stopped by addition of 50 µL chloroform, and the glycans were washed with water until the solution reached neutrality [18]. Permethylated glycan samples (0.5 µL) were applied to a ground steel target and mixed with 0.5 µL matrix (sDHB 10 µg/µL in 10 % aqueous acetonitrile). MALDI‐TOF‐MS spectra were acquired in the positive reflectron mode using an Ultraflex III TOF/TOF mass spectrometer (Bruker Daltonics, Bremen, Germany) equipped with a smartbeam laser (337 nm) and a LIFT‐MS/MS cell. Data were recorded over a mass range of *m/z* 800–5000 Da with an accelerating voltage of 25 kV. External calibration was performed using a glucose ladder ranging from *m/z* 851.2 Da (Glc_5_) to *m/z* 3768.2 Da (Glc_23_).

FlexAnalysis (Bruker Daltonics, Bremen, Germany) was used for baseline correction and peak picking. Signals with an *S*/*N* <6 were excluded from further analysis. The total area of all *N‐*glycans comprising the full isotopic envelope was normalized to 100% for each sample and used for relative quantification. Structural assignments were performed manually based on molecular ion composition, our previously reported datasets on MSCs [12], knowledge of biosynthetic pathways, and MALDI‐TOF/TOF fragmentation patterns. The peaks at *m/z* 2792.3, 3241.6, 3602.7, and 3963.9 were excluded from the analysis as they could be stemming from FCS (Figures S1). Cartoons were generated with Glycoworkbench [19].

### 2.5 Desialylation, APTS‐Labeling, and CE Coupled With Laser‐Induced Fluorescence (CE‐LIF)

Dried *N*‐glycans were dissolved in 40 µL of acetic acid solution (0.5 M) and incubated at 80°C for 3 h. Desialylated *N*‐glycans were subsequently desalted using self‐made graphite micro‐columns (Grace, Deerfield, IL, USA) [20, 21]. The micro‐columns were first pre‐equilibrated with 3 × 40 µL of 80% ACN containing 0.1% TFA, followed by 3 × 40 µL of 0.1% aqueous TFA. Samples were then applied, and the microcolumns were washed with 3 × 40 µL of 0.1% aqueous TFA. Desalted *N*‐glycans were eluted with 50% ACN containing 0.1% TFA and finally dried by centrifugal evaporation. Samples were labeled with APTS overnight at 37°C, as previously reported [20, 21]. Finally, the samples were diluted with 21 µL of water and analyzed by CE‐LIF.

CE‐LIF measurements were performed on a Beckman P/ACE MDQ system equipped with LIF detection (λex = 488 nm, λem = 510±10 nm) (Beckman Coulter, Fullerton, CA, USA). Separations were achieved by reversed polarity using a polyvinyl alcohol (PVA)‐coated capillary (50 nm id, 40 cm effective length to the window, 50.2 cm total length) using an acetate buffer (25 mM, pH 4.75) containing 0.4 % polyethylenoxide. The capillary was first rinsed with buffer for 2 min at 30 psi. Samples were then injected at 0.5 psi for 4 s, and separations were carried out at 30 kV.

### 2.6 Statistical Analysis

Statistical analyses were performed using IBM SPSS Statistics (version 25.0; SPSS Inc., Chicago, IL, USA). All variables were normalized by transformation to *z*‐scores. Data distributions and potential outliers were visually assessed using boxplots. Due to the low sample size within individual groups (*n* < 5), associations between variables were further evaluated using Spearman's rank correlation coefficient (*ρ*). Exact *p*‐values were calculated using a permutation approach with 5000 iterations. Two‐tailed *p*‐values ≤ 0.05 were considered statistically significant. *N*‐Glycosylation traits exhibiting strong positive or negative correlations (*ρ* ≥ 0.8 or *ρ* ≤ −0.8) were visualized using boxplots, *p*‐values are given in the Figure legends.

## 3 Results

**FIGURE 1:**
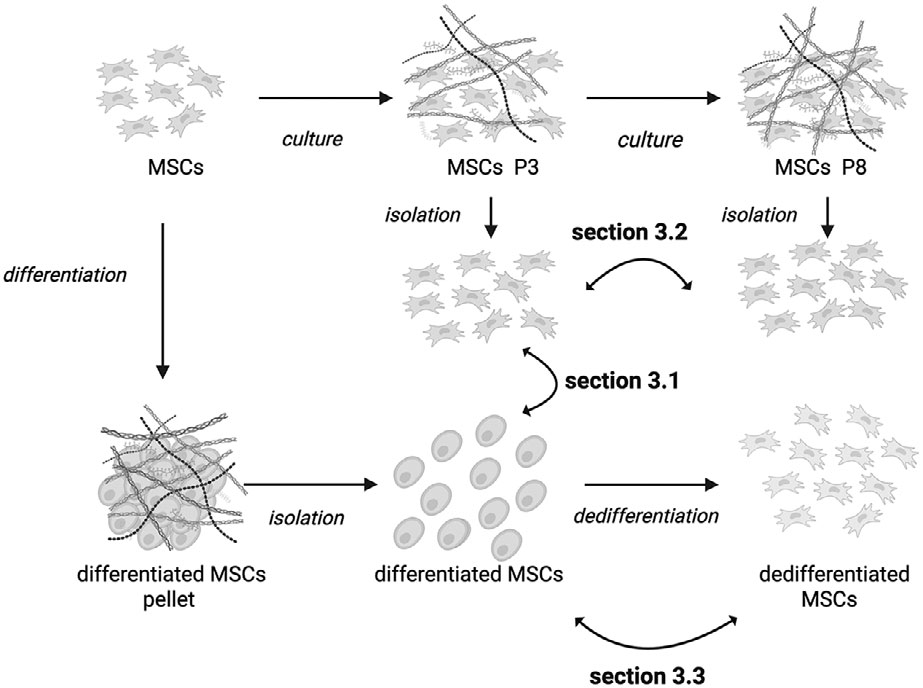
Overview of the MSCs cultured in this study. Arrows indicate what MSCs were compared in terms of *N‐*glycome in the Results section, and the numbers of the subsections are indicated in the Figure. The picture was created with BioRender.com.

Human MSCs were isolated from bone marrow aspirates; they subsequently underwent chondrogenic differentiation and dedifferentiation, respectively, as previously described [14] (Figures S2–S5). Figure 1 provides an overview of the various MSCs and their differentiated progenies, which were cultured, harvested, and analyzed for their *N*‐glycome.

### 3.1 *N*‐Glycome of MSCs and Its Changes During Chondrogenic Differentiation

We investigated *N*‐glycosylation changes in MSCs during chondrogenic differentiation. To this end, 10 different MSC preparations isolated from 10 distinct donors were utilized in this section. The *N*‐glycosylation profiles of undifferentiated MSCs (*n* = 4), as well as early‐stage (day 5, *n* = 3) and late‐stage (day 28, *n* = 3) chondrogenically differentiated MSCs, were investigated following their isolation from their extracellular matrix.

**FIGURE 2:**
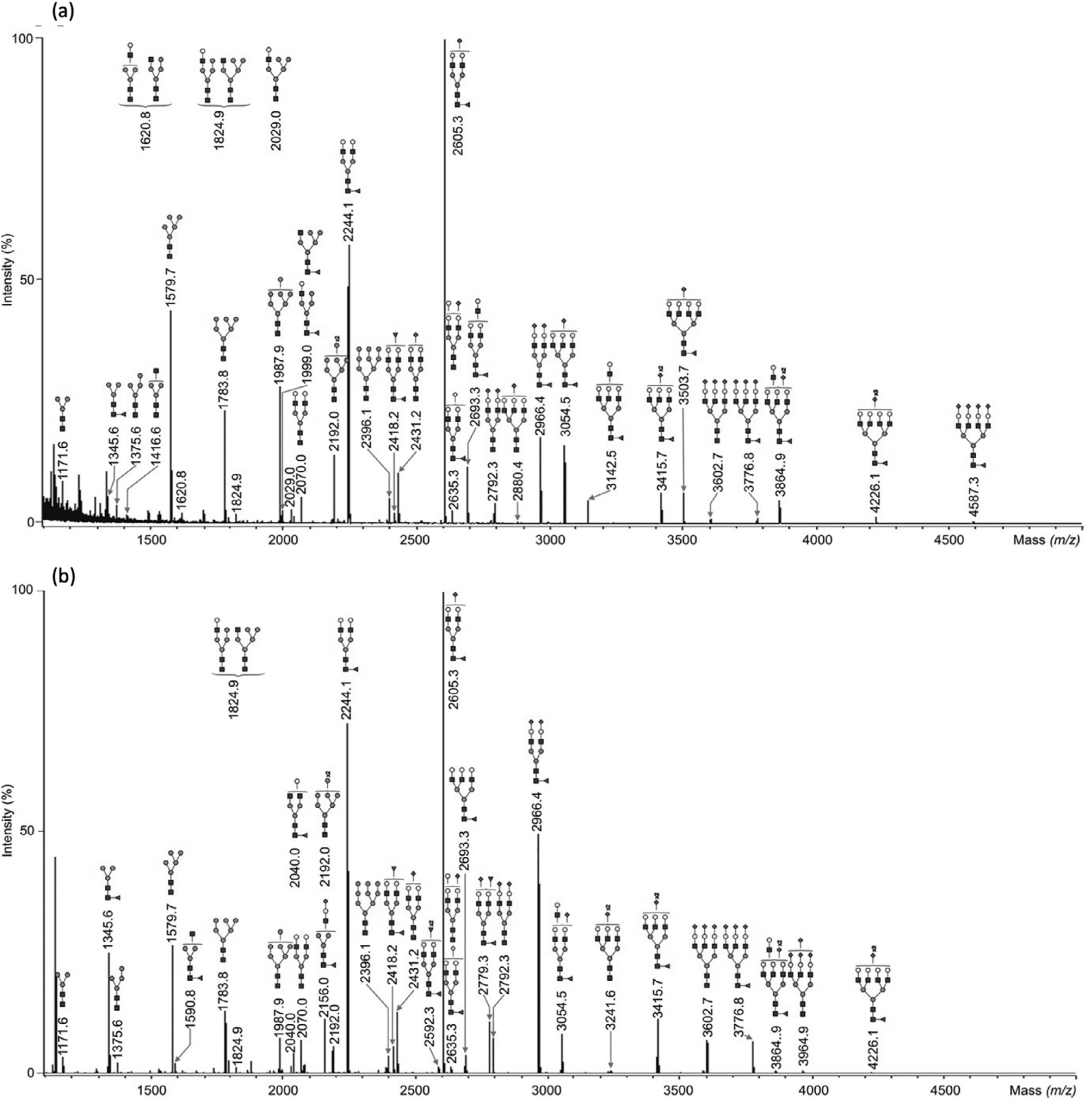
Representative MALDI‐TOF mass spectra of PNGase F‐released *N*‐glycans of (a) undifferentiated and (b) chondrogenically differentiated human MSCs (day 28) are shown together with the 30 most abundant structures. Day 28 chondrogenically differentiated MSCs were isolated from their ECM prior to *N*‐glycan analysis. Undifferentiated MSCs were cultured for three passages prior to the release of cell surface glycoproteins with trypsin. *N*‐Glycans were enzymatically cleaved from glycopeptides with PNGase F, permethylated, and measured by MALDI‐TOF‐MS. The black square represents *N*‐acetylglucosamine, the gray circle mannose, the white circle galactose, the black triangle fucose, and the black diamond *N*‐acetylneuraminic acid.

Cell surface (glyco)proteins were enzymatically released with trypsin, yielding glycopeptides. *N*‐Glycans, cleaved using PNGase F, were isolated using C18 cartridges and purified via carbograph columns. Finally, samples were permethylated and measured by means of MALDI‐TOF‐MS. Representative mass spectra of the resulting *N*‐glycosylation profiles for both undifferentiated and day 28 chondrogenically differentiated MSCs are shown in Figure 2.

Mass spectrometric profiling revealed the presence of high‐mannose, paucimannose, complex‐type, and hybrid *N*‐glycans. Core‐fucosylated biantennary complex‐type *N*‐glycans (*m/z* 2244.1, 2605.3, and 2966.4) as well as the high‐mannose N5N2 (*m/z* 1579.7) dominated the *N*‐glycan profiles of undifferentiated MSCs and chondrogenically differentiated MSCs at both day 5 and day 28. Fucosylated triantennary and tetraantennary *N*‐glycans were also detected. A total of 68 different *N*‐glycan signals were identified by MALDI‐TOF‐MS (Table S1), and their structures were verified using an array of exoglycosidases (Table S2). The most abundant *N*‐glycan signals were further analyzed by MALDI‐TOF/TOF mass spectrometry (Figure S6 and Table S3).

Poly‐*N*‐acetyl lactosamine (LacNAc) motifs decreased during chondrogenic differentiation; the presence of LacNAc motifs was confirmed by MALDI‐TOF/TOF with a diagnostic fragment ion H2N2 at *m/z* 935.3 (Figure S6A) in the H7N6F1 (*m/z* 3142.5) precursor. This fragment was absent from the MALDI‐TOF/TOF spectrum of MSCs at day 28 (data not shown). Monofucosylated *N*‐glycans were core‐fucosylated, as determined from the diagnostic fragments *at m/z* 474.0 (N1F1) and 719.1 (N2F1) (Figure S6A). Furthermore, bifucosylated *N*‐glycans additionally carried a Lewis^X^ motif (diagnostic fragment at *m/z* 660.1) as exemplified by the MALDI‐TOF/TOF mass spectrum of H5N4F2 (*m/z* 2418.2) in Figure S6B.

#### 3.1.1 Evaluation of Glycosylation Traits

**FIGURE 3:**
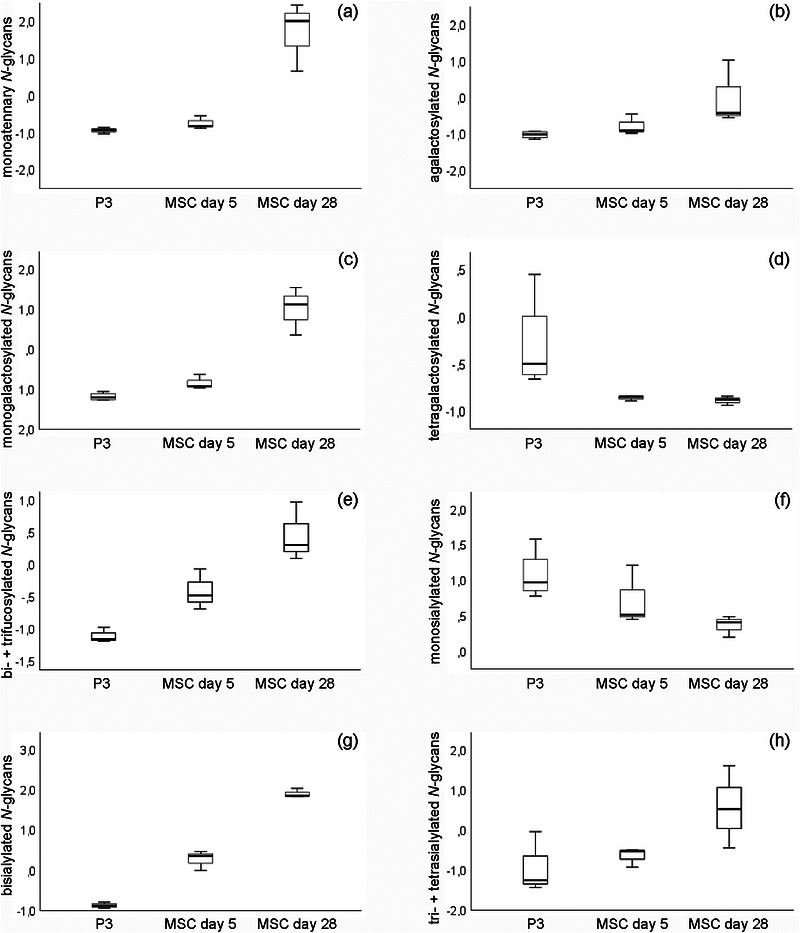
Boxplots comparing the glycosylation traits of undifferentiated MSCs at passage 3 with MSCs undergoing chondrogenic differentiation (day 5 and day 28). *N*‐Glycosylation traits showing a strong positive or negative correlation (*ρ* ≥ 0.8 or *ρ* ≤ −0.8) and an exact *p*‐value < 0.05 are shown. Due to the low sample size within individual groups (*n* < 5), associations between variables were additionally evaluated using Spearman's rank correlation coefficient (*ρ*). Exact *p*‐values were calculated using a permutation approach with 5,000 iterations. A two‐tailed *p*‐value < 0.05 was considered statistically significant. The following statistically significant glycosylation traits are shown: (a) monoantennary *N*‐glycans, (b) agalactosylated *N*‐glycans, (c) monogalactosylated *N*‐glycans, (d) tetragalactosylated *N*‐glycans, (e) bi + trifucosylated *N*‐glycans, (f) monosialylated *N*‐glycans, (g) bisialylated *N*‐glycans, (h) tri + tetrasialylated *N*‐glycans.

The total area of all detected *N‐*glycans was normalized to 100% for each sample and subsequently used for relative quantification across biological replicates. Assigned *N*‐glycans were grouped according to glycan structural features, including type (paucimannose, high‐mannose, hybrid, and complex), antennarity, galactosylation, fucosylation, and sialylation (Table S4). Descriptive statistics were used to summarize the data, which are presented as appropriate measures of central tendency and variability for all *N*‐glycosylation traits (Table S5). Associations between *N*‐glycosylation traits and MSC differentiation were assessed by fitting linear regression models, with a separate model generated for each glycosylation trait. The proportions of paucimannose, high‐mannose, hybrid, and complex‐type *N*‐glycans remained stable during chondrogenic differentiation (data not shown). Boxplots of statistically significant glycosylation traits are shown in Figure 3. The relative abundance of complex‐type monoantennary *N*‐glycans increased in MSCs differentiated for 28 days compared with undifferentiated MSCs and those at day 5 (Figure 3a). Agalactosylation and monogalactosylation were elevated in MSCs after 28 days of chondrogenic differentiation, whereas tetragalactosylation decreased (Figure 3b–d). Bi‐ and trifucosylation increased with MSC differentiation (Figure 3e). Regarding sialylation, an increase in bi‐, tri‐, and tetrasialylation was accompanied by a decrease in monosialylation over the course of chondrogenic differentiation (Figure 3f–h).

3.1.2

A portion of the non‐derivatized *N*‐glycan samples (undifferentiated MSCs, and MSCs at day 5 and day 28 of chondrogenic differentiation) was desialylated, labeled with APTS, and analyzed by CE‐LIF analysis. High‐mannose *N*‐glycans were identified, namely H5N2 (6.8 GU) and H6N2 (7.7 GU) (Figure S6). The relative abundances of complex‐type *N*‐glycans, such as the tetraantennary tetragalactosylated monofucosylated species H7N6F1 (13.8 GU), decreased on day 28 of chondrogenically differentiated cells. This corroborates the decrease in tetragalactosylated *N*‐glycans observed via MALDI‐TOF‐MS (Figure 3d).

### 3.2 Influence of Passage Number on *N*‐glycosylation of MSCs

**FIGURE 4:**
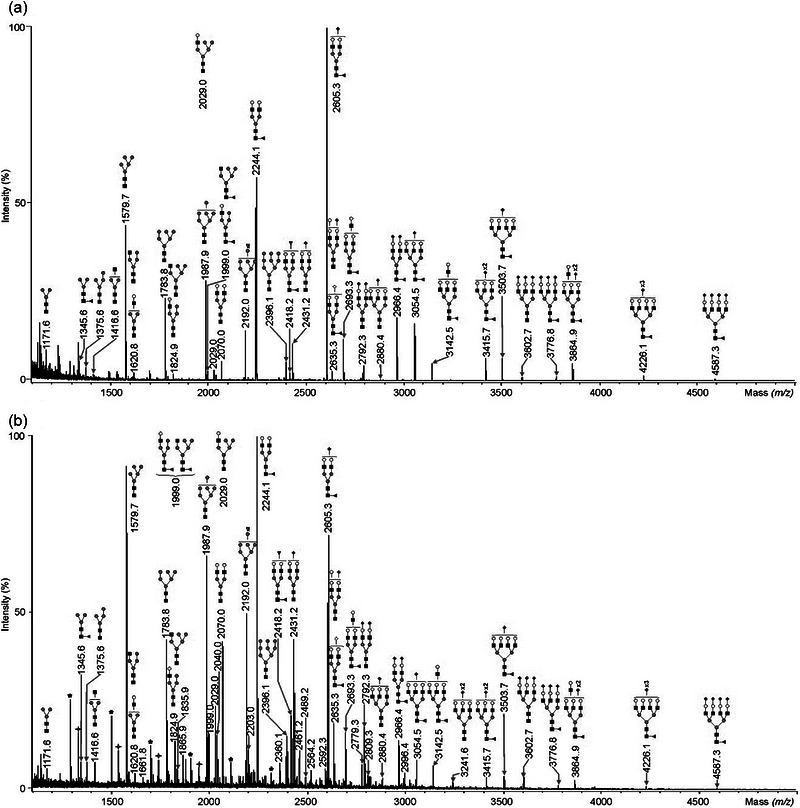
MALDI‐TOF mass spectra of PNGase F‐released *N*‐glycans of undifferentiated MSCs at different passages. A representative mass spectrum of undifferentiated MSCs at passage 3 (a) and passage 8 (b) is shown together with the 40 most abundant structures. Cell surface glycoproteins were directly digested from the cells with trypsin. *N*‐Glycans were enzymatically cleaved from glycopeptides with PNGase F, permethylated, and measured by MALDI‐TOF‐MS. The black square represents *N*‐acetylglucosamine, the gray circle mannose, the white circle galactose, the black triangle fucose, and the black diamond *N*‐acetylneuraminic acid. *Polyhexose contaminations. Non identified peaks.

In order to investigate the effect of passage number on the *N*‐glycosylation profile, MSCs were collected at passage 3 (*n* = 4 biological replicates) and 8 (*n* = 3 biological replicates). *N*‐Glycans were released from cell‐surface glycopeptides and measured by MALDI‐TOF‐MS (Figure 4). Mean values and SD of *N*‐glycosylation traits are shown in Table S5. Associations between *N*‐glycosylation traits and MSC aging were assessed by fitting linear regression models, with a separate model generated for each glycosylation trait. Selected boxplots of the glycosylation traits are shown in Figure S8. An increase in paucimannose (Figure S8a) and hybrid‐type (Figure S8b) *N*‐glycans was observed after eight passages. Regarding complex‐type *N*‐glycans, increases in bi‐, tri‐, tetrasialylation (Figure S8k–l) and decreases in biantennarity (Figure S8d), bigalactosylation (Figure S8g), and core‐fucosylation (Figure S8i) were detected.

### 3.3 *N*‐Glycosylation Profile of Chondrogenic Dedifferentiated Cells

**FIGURE 5:**
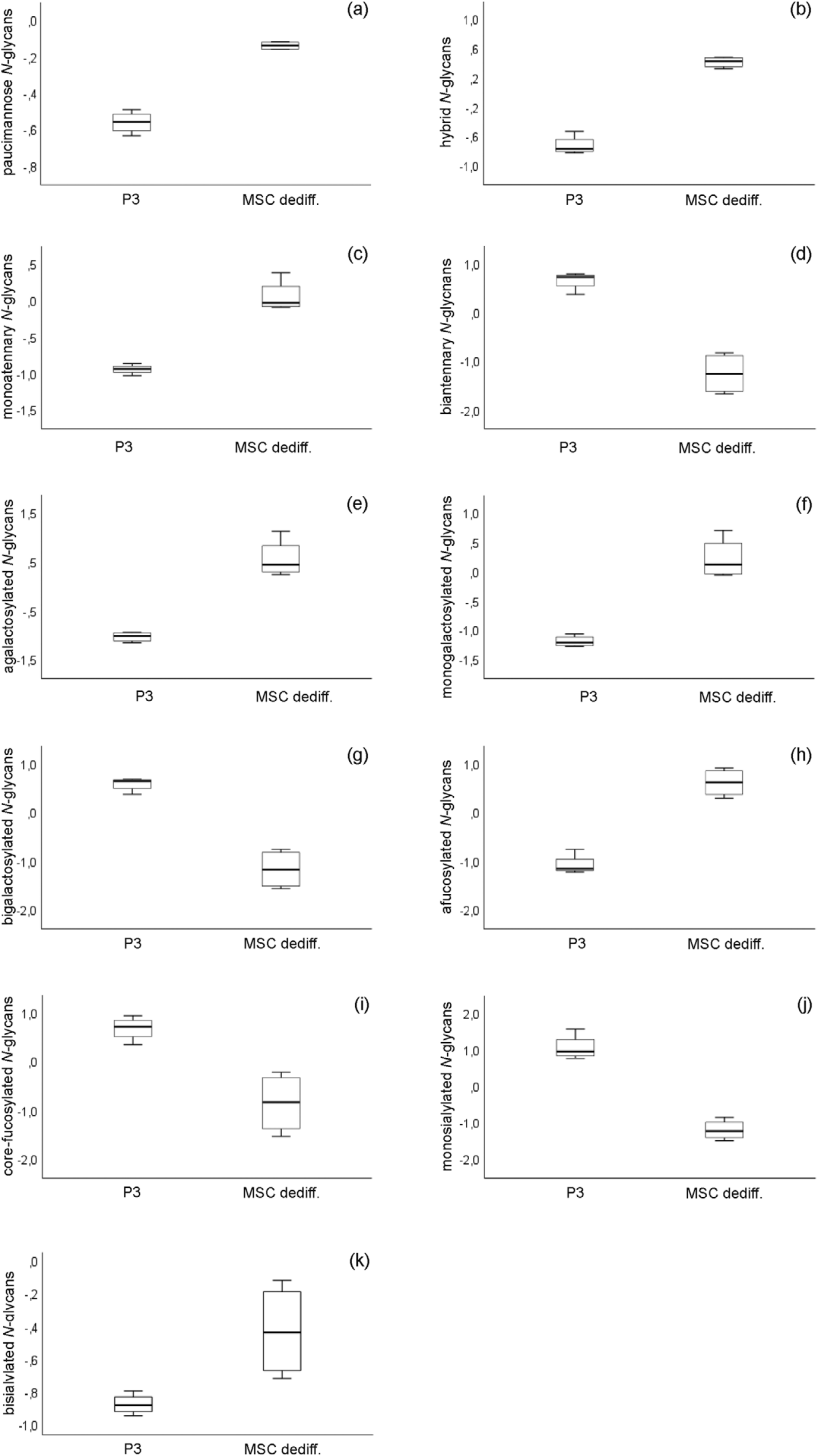
Boxplots comparing the *N*‐glycosylation traits of undifferentiated MSCs with dedifferentiated MSCs *N*‐Glycosylation traits showing a strong positive or negative correlation (*ρ* ≥ 0.8 or *ρ* ≤ −0.8) and an exact *p*‐value < 0.05 are shown. Given the low sample size within individual groups (*n* < 5), associations between variables were additionally evaluated using Spearman's rank correlation coefficient (*ρ*). Exact *p*‐values were calculated using a permutation approach with 5000 iterations. A two‐tailed *p*‐value < 0.05 was considered statistically significant. Traits meeting this criterion are shown: (a) paucimannose *N*‐glycans, (b) hybrid *N*‐glycans, (c) monoantennary *N*‐glycans, (d) biantennary *N*‐glycans, (e) agalactosylated *N*‐glycans, (f) monogalactosylated *N*‐glycans, (g) bigalactosylated *N*‐glycans, (h) afucosylated *N*‐glycans, (i) core‐fucosylated *N*‐glycans, (j) monosialylated *N*‐glycans, (k) bisialylated *N*‐glycans.

Dedifferentiation of chondrogenically differentiated cells gives rise to MSCs that are comparable to undifferentiated MSCs as judged by phase contract microscopy, flow cytometry, and qPCR (Figure S2 and S5). The *N*‐glycosylation profile of chondrogenically dedifferentiated MSCs (*n* = 4 biological replicates) was compared to that of undifferentiated MSCs (*n* = 4 biological replicates). Dedifferentiated MSCs were obtained as follows: each MSC sample, stemming from a single donor, was first chondrogenically differentiated for 28 days and then dedifferentiated into MSCs in FCS‐containing medium for an additional 28 days. Contamination peaks stemming from FCS were particularly pronounced in dedifferentiated cells, despite carefully washing the cells four times with PBS after harvesting. This is likely attributable to the prolonged culture period. Representative MALDI‐TOF mass spectra of undifferentiated MSCs and chondrogenically dedifferentiated MSCs are presented in Figure S9a,b, respectively. Mean values and SD of *N*‐glycosylation traits for undifferentiated and dedifferentiated MSCs are presented in Table S5. Associations between *N*‐glycosylation traits and MSC dedifferentiation were assessed by fitting linear regression models, with a separate model generated for each glycosylation trait. Boxplots comparing the glycosylation traits of undifferentiated MSCs with dedifferentiated MSCs are presented in Figure 5. Paucimannose (Figure 5a) and hybrid‐type *N*‐glycans (Figure 5b) increased upon dedifferentiation. Decreases in bigalactosylation (Figure 5g) and core‐fucosylation (Figure 5i) were accompanied by an increase in bisialylation (Figure 5k) upon dedifferentiation.

## 4 Discussion

In this work, we characterized the *N*‐glycosylation profile of undifferentiated MSCs, chondrogenic differentiated MSCs and dedifferentiated MSCs. We also investigated the influence of the passage number on the *N*‐glycosylation profile of undifferentiated MSCs.

Differentiated MSCs were found to contain high‐mannose as well as complex‐type *N*‐glycans, which is in accordance with glycome data published by different groups for human primary chondrocytes [22, 23, 24]. Toegel et al. [24] analyzed the carbohydrate structures of the glycocalyx of primary human chondrocytes using a panel of lectins, including *Lens culinaris* agglutinin and *Sambucus nigra* agglutinin, confirming the presence of high‐mannose *N*‐glycans and α(2‐6)‐linked sialic acids in chondrocytes [24]. Pabst et al. studied the *N*‐glycosylation of primary human chondrocytes by LC‐MS and showed the presence of high‐mannose *N*‐glycans and complex‐type biantennary *N*‐glycans that were mostly α(2‐6)‐sialylated [23]. They also confirmed by qPCR that α(2‐6)‐sialyltransferases were overexpressed in primary chondrocytes [23]. Moreover, Bernard and coworkers identified fibronectin as a source of high‐mannose *N*‐glycans in chondrocytes [22].

Complex‐type monoantennary *N*‐glycans increased after chondrogenic differentiation of MSCs, indicating a decrease in antennarity. Our data are in accordance with the study by Yang et al. [25] on the biosynthetic pathway of human chondrocytes isolated from osteoarthritis patients. Indeed, they demonstrated the activity of GlcNAc‐transferases I and II as well as β4Gal‐transferase. In addition, the activities of GlcNAc‐transferases IV and V were below detectable levels [25], suggesting a significantly lower amount of tri‐ and tetraantennary *N*‐glycans in human chondrocytes. In addition, these results are also in line with studies by Richard et al. [26] who investigated glycosyltransferase activities in chondrocytes from patients with osteoarthritis and normal human cartilage. Glycosyltransferase genes were also quantified in chondrocytes from osteoarthritis patients at the mRNA level using RT‐qPCR. MGAT1 and MGAT2, the genes responsible for GlcNAc‐transferases I and II, respectively, were expressed at higher levels than *MGAT4* and *MGAT5*, the genes responsible for GlcNAc‐transferases IV and V [27].

In this study, we found a decrease in complex‐type monosialylated *N*‐glycans accompanied by increases in bi‐, tri‐, and tetrasialylated *N*‐glycans during chondrogenic differentiation. Due to the sample preparation method we employed, our analysis of sialylation was limited to detecting their presence, without distinguishing between α(2‐3)‐ and α(2‐6)‐linked sialic acids. Notably, sialic acid linkages have been analyzed by other groups using lectin staining of the cell surface glycocalyx. The cell surface glycocalyx of MSCs is α(2‐3)‐sialylated rather than α(2‐6)‐sialylated as judged from *Maackia amurensis* (MAA) and *S. nigra* agglutinin (SNA) stainings [28]. In contrast, in primary chondrocytes, SNA staining of α(2‐6)‐linked sialic acids was higher than MAA staining [25, 27]. Similar results were observed at the mRNA level, where the expression of *ST6Gal1* (which transfers α(2‐6)‐linked sialic acids on *N*‐glycans) was higher than that of *ST3Gal4* (responsible for α(2‐3)‐linked sialylation of *N*‐glycans).

LacNAc epitopes were found in this work in undifferentiated MSCs but were absent from day 28 chondrogenically differentiated cells. This is in agreement with Heiskanen et al., who also performed *Solanum tuberosum* lectin to recognize linear poly‐LacNAc chains [28].

We were the first to study MSCs senescence from a glycomics perspective by comparing the *N*‐glycome of early‐ and late passage bone marrow MSCs. Our glycosylation analysis indicates that certain *N‐*glycan patterns are associated with the aging of MSCs. Specifically, we found an increase in paucimannose and hybrid‐type *N*‐glycans in MSCs cultured for eight passages. In addition, sialylation increased, whereas antennarity, galactosylation, and fucosylation decreased over the course of passaging. High‐mannose *N*‐glycans were not statistically different upon senescence. In contrast, Wang and coworkers observed a decrease in high‐mannosylation of umbilical MSCs likely due to a different cell source and preparation protocol. Indeed, these authors performed reduction followed by glycoprotein alkylation prior to tryptic digestion [29]: Consequently, the two studies cannot be directly compared.

We were also the first group to report the *N*‐glycome profile of MSCs upon dedifferentiation. The main changes observed included a decrease in bigalactosylation, core‐fucosylation, as well as an increase in paucimannosylation, hybrid‐type, and bisialylation upon dedifferentiation in FCS‐containing medium. Changes in tri‐ and tetrasialylation were not statistically significant, which may be due to the low number of biological replicates. In contrast, Templeton et al., used glycoengineering to reduce sialylation in an ischemia model, thereby preserving the therapeutic potential of stem cells [30]. Interestingly, these authors showed that MSCs sialylation can be regulated by external stimuli such as IFN‐gamma or exposure to culture media low in FBS, most likely by regulating the transcription of the corresponding sialyltransferases [30].

Finally, based on the data presented in this study, although the sample size was small, the *N*‐glycosylation profile appears to reflect the differentiation stage of MSCs and could potentially be used for quality control of stem cells. However, larger studies are required to confirm these findings.

## Author Contributions

Conceptualization, V.B., R.T., and M.S; methodology, investigation, and data analysis, H.M. and K.B.; writing – original draft preparation, H.M. and V.B.; writing – review and editing, all authors; supervision and project administration, V.B. All authors have read and agreed to the published version of the manuscript.

## Funding

This research was funded by Investitionsbank Berlin and the European Regional Development Fund (grants no.: 10147244 and 10147246).

## Conflicts of Interest

The authors declare no conflicts of interest.

## Supporting information



Figure S1: MALDI‐TOF mass spectrum of FBS used for MSC culture in this study. Figure S2: Characterization of undifferentiated MSCs, chondrogenic differentiated cells MSCs at day 28 and dedifferentiated MSCs. (a) Phase contrast microscopy: fibroblast‐like morphology of undifferentiated MSCs (upper panel), chondrogenic differentiated MSCs at day 28 (middle panel) and dedifferentiated MSCs (lower panel). Flow cytometric analysis showing (b) cell surface markers and (c) cell size. GraphPad Prism4 (GraphPad Software) was used for drawing graphs. Figure S3: (a) Chondrogenic pellets (upper panel) used to isolate chondrogenic differentiated MSCs (day 28) (middle panel). Chondrogenic differentiated MSCs (day 28) were dedifferentiated, yielding dedifferentiated MSCs (lower panel). (b) The chondrogenic potential of chondrogenic differentiated MSCs was checked by positive stainings with Alcian blue, toluidine, safranin O, and H&E. Chondrogenic ability was further confirmed by immunostaining for collagen type I, collagen type II, COL2A1, aggrecan, and collagen type X. Figure S4: Chondrocytes isolated from native cartilage were stained for collagen type I, II, type 2A1, aggrecan, collagen X, Safranin O expressions and toluidine blue. These chondrocytes were used as the positive control for the experiments shown in Figure S3. Figure S5: qPCR analysis of four genes that are of relevance for chondrogenic differentiation, namely (a) COL2A1, (b) ACAN, (c) SOX 9 and (d) COL1A1. The chondrogenic differentiation was confirmed by an upregulated expression of these genes when compared with negative controls and undifferentiated MSCs. Dedifferentiation was confirmed by a statistically significant downregulation of the four genes. Experiments were performed in triplicate. Student´s t‐test was performed for statistical analysis, and asterisks were assigned in the order p** < 0.01, and p*** < 0.001; mean ± SEM. Figure S6. MALDI‐TOF/TOF mass spectrum of *m/z* 3142.5 (H7N6F1) derived from undifferentiated MSCs (a) and *m/z* 2418.2 (H5N4F2) derived from day 28 chondrogenically differentiated MSCs (b). Figure S7. CE‐LIF electropherograms of PNGase F‐released and desialylated *N*‐glycans from **(a)** undifferentiated, **(b)** day 5 and **(c)** day 28 chondrogenically differentiated human MSCs. Cells were digested with trypsin in order to release cell surface (glyco)peptides. (Glyco)peptides were subjected to PNGase F digestion to release *N*‐glycans, desialylated and measured by CE‐LIF. Blue square represents *N*‐acetylglucosamine, green circle mannose, yellow circle galactose, red triangle fucose. Migration times are presented as glucose units because mobility varies from run to run as the electrolyte concentration changes [20, 21]. Figure S8. Boxplots comparing *N*‐glycosylation traits of undifferentiated MSCs at passage 3 and passage 8. Figure S9. MALDI‐TOF‐MS of PNGase F‐released *N*‐glycans. Representative mass spectra of undifferentiated MSCs (a) and chondrogenic dedifferentiated MSCs (b) are shown together with the 55 most abundant structures. Table S1. Average relative abundances of PNGase F–released *N*‐glycans from undifferentiated MSCs and chondrogenically differentiated human MSCs at day 5 and day 28 of differentiation, derived from three independent donors. Table S2. Exoglycosidase digestions of PNGase F‐released *N*‐glycans from undifferentiated MSCs and MSCs chondrogenically differentiated for 28 days. Table S3. List of *N*‐glycans precursors fragmented by MALDI‐TOF/TOF‐MS; diagnostic fragments ions are indicated in the Table. Table S4. The assigned *N*‐glycans were grouped according to glycan structural features: type (paucimannose, high‐mannose, hybrid, complex) and glycosylation traits (antennarity, galactosylation, fucosylation and sialylation). Table S5. Descriptive statistics were used to summarize the data and are presented as appropriate measures of central tendency and variability for all *N*‐glycosylation traits (Table S4). SE, standard error; SD, standard deviation.**Supporting File 1**: pmic70124‐sup‐0001‐SuppMat.pdf.

**Supporting File 2**: pmic70124‐sup‐0002‐Figures.pdf.

**Supporting File 3**: pmic70124‐sup‐0003‐Tables.pdf.

## Data Availability

The raw data is available on Glycopost with accession number GPST000628.
