# Supplementary material for: The N‐Glycome to Differentiate Mesenchymal Stem Cells Upon Chondrogenic Differentiation, Dedifferentiation, and Senescence
Source: Proteomics. 2026 Mar 25;26(7):17–27. doi: 10.1002/pmic.70124 (PMC13327702; doi:10.1002/pmic.70124)
Supplement: Supplementary file 1 — Figure S1: MALDI‐TOF mass spectrum of FBS used for MSC culture in this study. Figure S2: Characterization of undifferentiated MSCs, chondrogenic differentiated cells MSCs at day 28 and dedifferentiated MSCs. (a) Phase contrast microscopy: fibroblast‐like morphology of undifferentiated MSCs (upper panel), chondrogenic differentiated MSCs at day 28 (middle panel) and dedifferentiated MSCs (lower panel). Flow cytometric analysis showing (b) cell surface markers and (c) cell size. GraphPad Prism4 (GraphPad Software) was used for drawing graphs. Figure S3: (a) Chondrogenic pellets (upper panel) used to isolate chondrogenic differentiated MSCs (day 28) (middle panel). Chondrogenic differentiated MSCs (day 28) were dedifferentiated, yielding dedifferentiated MSCs (lower panel). (b) The chondrogenic potential of chondrogenic differentiated MSCs was checked by positive stainings with Alcian blue, toluidine, safranin O, and H&E. Chondrogenic ability was further confirmed by immunostaining for collagen type I, collagen type II, COL2A1, aggrecan, and collagen type X. Figure S4: Chondrocytes isolated from native cartilage were stained for collagen type I, II, type 2A1, aggrecan, collagen X, Safranin O expressions and toluidine blue. These chondrocytes were used as the positive control for the experiments shown in Figure S3. Figure S5: qPCR analysis of four genes that are of relevance for chondrogenic differentiation, namely (a) COL2A1, (b) ACAN, (c) SOX 9 and (d) COL1A1. The chondrogenic differentiation was confirmed by an upregulated expression of these genes when compared with negative controls and undifferentiated MSCs. Dedifferentiation was confirmed by a statistically significant downregulation of the four genes. Experiments were performed in triplicate. Student´s t‐test was performed for statistical analysis, and asterisks were assigned in the order p** < 0.01, and p*** < 0.001; mean ± SEM. Figure S6. MALDI‐TOF/TOF mass spectrum of m/z 3142.5 (H7N6F1) derived from undiffer [file PMIC-26--s002.pdf]

Supplementary document: MSC isolation, authentication, and chondrogenic differentiation

1. Materials and Methods

1.1. Culturing of MSCs

The 1-ml aspirate was seeded per T175 cm^2 of culture flasks (Becton Dickinson, Heidelberg, Germany). After 48 hours, non-adherent cells and cellular debris were washed away by media exchange, and cultures were further expanded in Dulbecco Modified Eagle Medium (DMEM; Biochrom, Berlin, Germany), supplemented with 10% fetal bovine serum (FBS; Hyclone, Cramlington, UK), 20 mM Hepes buffer (Biochrom), 2 mM L-glutamine (Biochrom), 2 ng/ml human basic-fibroblast growth factor (bFGF; Pepro Tech, London, UK), 100 units/ml penicillin and 100 µg/ml streptomycin (Biochrom), under established conditions. MSCs were further cultured and expanded in Dulbecco's Modified Eagle's Medium (Biochrom, Berlin, Germany) supplemented with 10% FBS, 1% penicillin/streptomycin, 1% glutamine, 2% HEPES, and 2 ng/mL of bFGF (Tebu-bio, Offenbach, Germany). Cultivated MSCs were triple negative for the markers CD45, CD34, and CD14, but stained positively for the markers CD73, CD105, CD106, CD90, and CD44 (see 2.3).

1.2. Chondrogenic differentiation, cell isolation and dedifferentiation

MSCs (2.5×10^5) were suspended in chondrogenic differentiation medium, consisting of DMEM (4.5 g/L glucose; Biochrom AG, Berlin, Germany), Insulin-Transferrin-Selenium (ITS) supplements 100 n*M* dexamethasone, 0.17 m*M* ascorbic acid-2-phosphate, 1 m*M* sodium pyruvate, 0.35 m*M* L-proline and 10 ng/ml transforming growth factor-β3 (TGF-β3; PeproTeck, Hamburg, Germany). MSCs were then

centrifuged (150 *g*, 5 minutes) to form high-density micromass culture pellets. MSCs were resuspended in 500 µl chondrogenic differentiation medium and chondrogenic differentiation was achieved by incubation for 28 days at 37°C. The medium was changed 3 times per week. Control pellets were cultured in the same medium in the absence of TGF-β3.

After 28 days, cells were isolated from chondrogenic pellets with 300 U of collagenase II, 20 U of collagenase P, and 2 m*M* $CaCl_2$ for 90 minutes at 37°C. Subsequently, some cells were cultured as a monolayer for 14 days in the presence of the chondrogenic differentiation-specific stimulus of TGF-β3, to maintain their chondrogenic nature. For comparison, another portion of the chondrogenically differentiated cells was cultured for five passages in normal MSCs expansion medium (DMEM (4.5 g/L glucose; Biochrom AG, Berlin, Germany), 10% FCS, ITS supplements 100 n*M* dexamethasone, 0.17 m*M* ascorbic acid-2-phosphate, 1 m*M* sodium pyruvate, 0.35 m*M* L-proline to accelerate proliferation and to generate dedifferentiated progenitor cells.

Chondrocytes were also isolated from native cartilage sections with 300 U of collagenase II, 20 U of collagenase P, and 2 m*M* $CaCl_2$ at 37°C by incubation overnight in the spinner flasks and were used as a control in all experimental procedures.

1.3. Flow-cytometric analysis and validation of surface markers

All cell types were prepared as single-cell suspension, then washed with PBS/0.5% bovine serum albumin (BSA; both Biochrom AG, Berlin, Germany), and centrifuged for 5 minutes at 250 *g*. The resuspended cells in cold PBS/0.5% BSA were incubated for 15 min on ice with R-phycoerythrin-labeled mouse anti-human CD14, CD34, CD73, CD166, and fluorescein isothiocyanate-labeled mouse anti-human CD44, CD45, CD90, and CD105 antibodies. All antibodies were purchased from BD-Pharmingen

(Heidelberg, Germany) except CD105, which was purchased from Acris Antibodies (Hiddenhausen, Germany).

After incubation, cells were centrifuged (250 *g*, 5 minutes), washed with cold PBS/0.5% BSA, and resuspended in the same buffer before cytometric analysis. To examine the surface proteins, staining was performed as described earlier [1]. Briefly, after fixation with 4% paraformaldehyde for 15 min, cells were permeabilized for 10 min with FACS permeabilizing solution-2 (Becton Dickinson, Germany). The propidium iodide (100 µg/ml) staining was applied for the exclusion of dead cells and cellular debris, whereas unstained cells were used as a negative control. The single-cell suspension was analyzed by flow cytometry, and CellQuest software (Becton Dickinson) was used for the interpretation and analysis of results.

1.4. Histology and immunohistochemistry

To examine native sections and chondrogenesis pellets, specimens were embedded in Tissue-Tek with O.C.T. compound (Sakura Finetek, Torrance, USA), and then were frozen in liquid nitrogen and cryosectioned (6-µm thickness). For cartilage-specific proteoglycan examination, sections were stained with Alcian blue 8GX (Roth, Karlsruhe, Germany) and counterstained with nuclear fast red. For imaging the deposition and accumulation of collagen type I, collagen type II, collagen type 2A1, aggrecan, collagen X in the extracellular matrix (ECM), cryosections (6 µm) were incubated for 1 hour with primary antibodies (Cell signaling Technology, Danvers, MA, USA). Subsequently, the sections were processed according to the manufacturer’s recommendation with the Envision system peroxidase kit (DAKO, Hamburg, Germany), followed by hematoxylin counterstaining (Merck, Darmstadt, Germany).

1.5. RNA isolation and qPCR

For RNA isolation, each cell type (MSCs, chondrogenic differentiated, and dedifferentiated cells) was separately mixed with TriReagent. Differentiated chondrogenic pellets from each individual donor were first pooled in a 2-ml Eppendorf tube, then mixed with TriReagent and mechanically homogenized with an Ultra-Turrax (IKA, Staufen, Germany) [2]. Then, 1-bromo-3-chloro-propane was added to all samples, followed by centrifugation (45 minutes, 13,000 *g*), and the upper phase, being free of proteins, was collected and mixed with an equal amount of ethanol. Subsequently, samples were processed with the RNeasy Mini Kit (Qiagen, Hilden, Germany), according to manufacturer´s recommendation. The quantity and quality of eluted RNA was ensured by NanoDrop measurement (NanoDrop Products, Wilmington, USA).

For qPCR, cDNA was synthesized from 2.5 µg total RNA by using the iScript cDNA synthesis kit (BioRad, Munich, Germany). TaqMan qPCR was executed in triplicates in 96-well optical plates on a Mastercycler ep Realplex2 S system (Eppendorf, Hamburg, Germany). The gene-expression assays for typical chondrogenic-specific genes were performed with TaqMan probes and primer sets (Applied Biosystems, Darmstadt, Germany). Quantitative gene expression was analyzed for collagen type 2 A1 (*COL2A1*; Hs 00264051_m1), SRY (sex-determining region Y)-box-9 (*SOX9*; Hs 00165814_m1), ACAN (*ACAN*; Hs 04982230_s1), collagen type 1 A1 (*COL1A1*; Hs 00164004_m1) and glyceraldehyde-3-phosphate dehydrogenase (*GAPDH;* Hs99999905_m1). The expression of *COL2A1, COL1A1, ACAN* and *SOX9* genes was normalized to the endogenous *GAPDH* expression level and calculated with the 2-ΔΔCt formula in percentage of *GAPDH* expression.

2. Results

Human MSCs were isolated from bone marrow aspirates, they subsequently underwent chondrogenic-differentiation (MSCs undifferentiated (n=4 biological replicates), MSCs day 5 (n=3), MSCs day 38 (n=3)) and dedifferentiation (n=4), respectively, as previously described [1].

MSCs, differentiated and dedifferentiated cells exhibited different morphologies as shown by phase contrast microscopy (Supplementary Material Figure S1a). MSCs and dedifferentiated cells revealed the typical fibroblast-like morphology in passage 3 (Supplementary Material Figure S1a). Flow cytometric analysis was performed to characterize the MSCs for their typical specific surface antigens, and to determine the expression of the following antigens in chondrogenic differentiated and dedifferentiated cells. The surface screening of MSCs showed a positive expression of CD44, CD73, CD90, CD105, and CD166, and a negative expression of CD14, CD34, and CD45 antigens in MSCs as well as in differentiated and dedifferentiated cells (Supplementary Material Figure S1b). Comparative flow-cytometric analysis showed higher expression of surface antigens in MSCs as compared to chondrogenic differentiated and dedifferentiated cells. Chondrogenic differentiated cells were smaller in size than MSCs and dedifferentiated cells (Figure S1c). Cryosections of the pellets (thickness 6 µm) from chondrogenic differentiated cells were positive for Alcian blue staining, toluidine staining, safranin o staining, and H&E staining reflecting presence of cartilage-specific proteoglycans (Supplementary Material Figure S2). The chondrogenic ability of these cells was further reflected by the positive expression of cartilage-specific collagen types I, II, 3A1, aggrecan and collagen X (Supplementary Material Figure S2). Chondrocytes isolated from native cartilage were used as controls (Supplementary Material Figure S3).

After chondrogenic differentiation, cells were released from the compact pellets enzymatically using collagenase II, collagenase P, and 2 m*M* $CaCl_2$. Released cells were cultivated in culture flasks to remove components of the extracellular matrix, and their differentiated state was maintained in the presence of the chondrogenic differentiation stimulus TGF-β3 for 14 days. Dedifferentiation was performed by five passages in normal MSCs expansion medium.

The chondrogenic potential of cultured cells showed a positive expression of collagen type I, II, aggrecan, and collagen X, (Supplementary Material Figure S2) compared with controls (Supplementary Material Figure S3).

On the gene level, the chondrogenic nature was verified by the expression of cartilage-specific genes *COL2A1, COL1A1, ACAN* and *SOX9*. All genes showed an upregulated expression in the chondrogenic samples compared with controls (Supplementary Material Figure S4). These results confirmed the well-advanced state of chondrogenic differentiation. Dedifferentiation was verified by a statistically significant downregulation of the four genes *COL2A1, COL1A1, ACAN* and *SOX9*.

[1] Ullah, M., Eucker, J., Sittinger, M., Ringe, J., Mesenchymal stem cells and their chondrogenic differentiated and dedifferentiated progeny express chemokine receptor CCR9 and chemotactically migrate toward CCL25 or serum. *Stem Cell Res Ther* 2013, *4*, 99.
[2] Chomczynski, P., A reagent for the single-step simultaneous isolation of RNA, DNA and proteins from cell and tissue samples. *BioTechniques* 1993, *15*, 532–534, 536–537.
