# Supplementary material for: The N‐Glycome to Differentiate Mesenchymal Stem Cells Upon Chondrogenic Differentiation, Dedifferentiation, and Senescence
Source: Proteomics. 2026 Mar 25;26(7):17–27. doi: 10.1002/pmic.70124 (PMC13327702; doi:10.1002/pmic.70124)
Supplement: Supplementary file 2 — Supporting File 2: pmic70124‐sup‐0002‐Figures.pdf. [file PMIC-26--s003.pdf]

Supplementary data

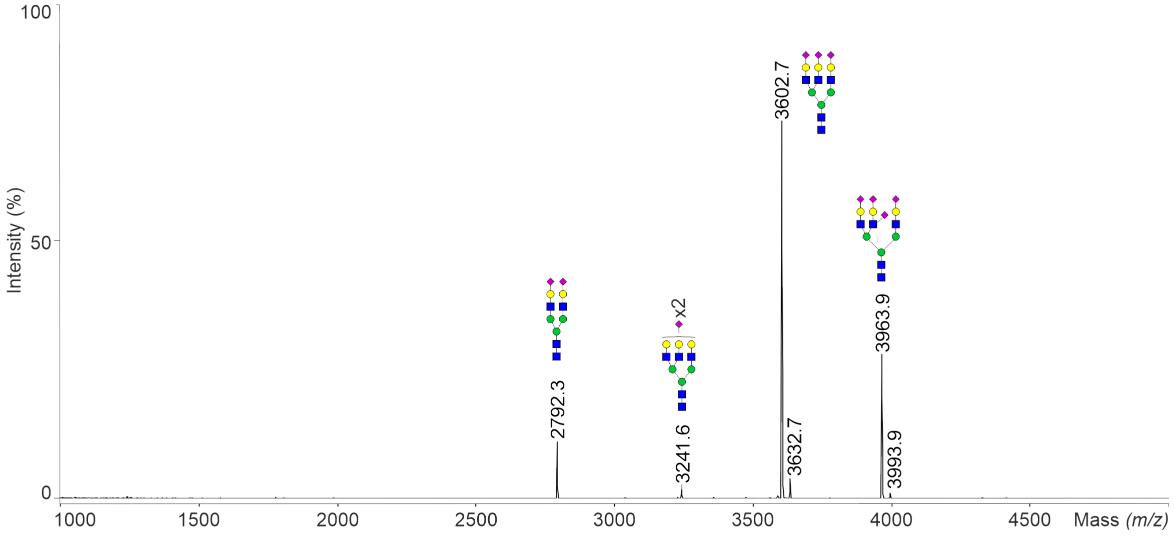

Figure S1: MALDI-TOF mass spectrum of FBS used for MSC culture in this study. Blue square represents *N*-acetylglucosamine, green circle mannose, yellow circle galactose, red triangle fucose and purple diamond *N*-acetylneuraminic acid.

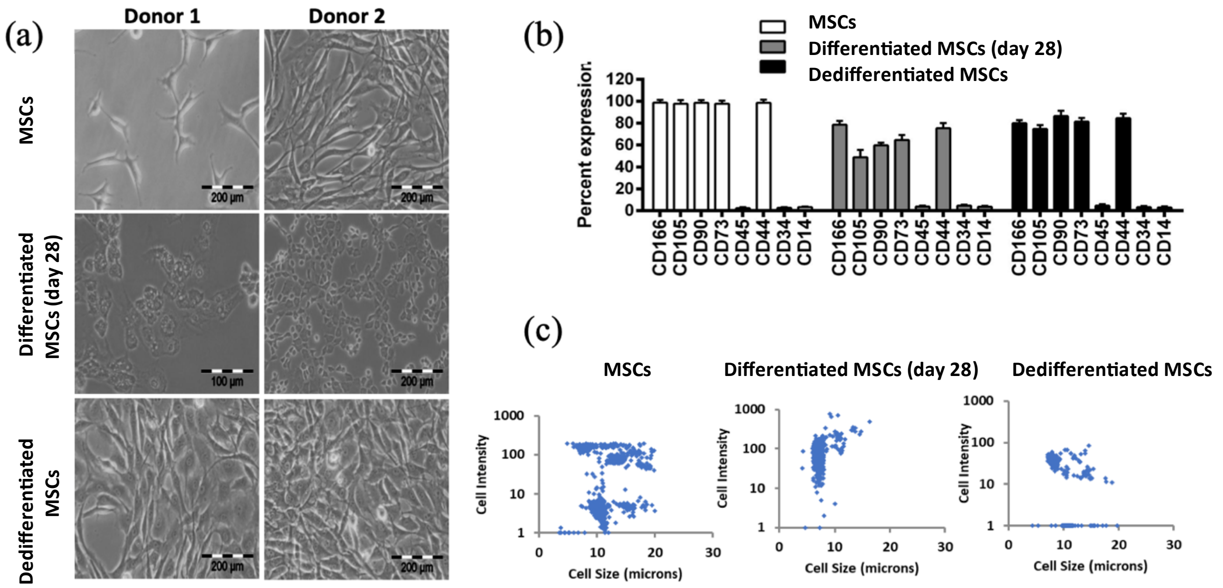

Figure S2: Characterization of undifferentiated MSCs, chondrogenic differentiated cells MSCs at day 28 and dedifferentiated MSCs. (a) Phase contrast microscopy: fibroblast-like morphology of undifferentiated MSCs (upper panel), chondrogenic differentiated MSCs at day 28 (middle panel) and dedifferentiated MSCs (lower panel). Flow cytometric analysis showing (b) cell surface markers and (c) cell size. GraphPad Prism4 (GraphPad Software) was used for drawing graphs.

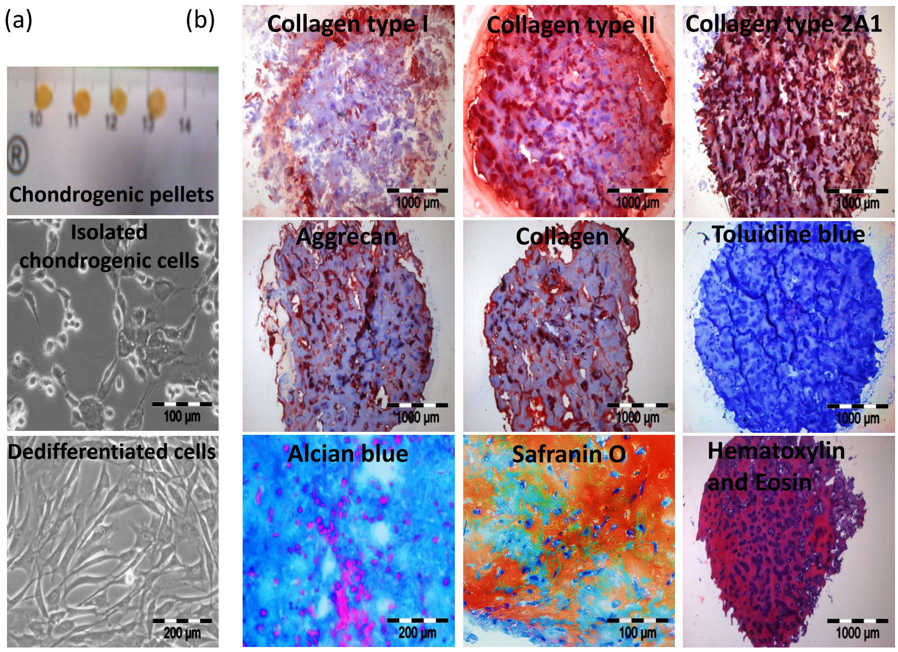

Figure S3: (a) Chondrogenic pellets (upper panel) from which chondrogenic differentiated MSCs (day 28) were isolated (middle panel). Chondrogenic differentiated MSCs (day 28) were dedifferentiated, yielding dedifferentiated MSCs (lower panel). (b) The chondrogenic potential of chondrogenic differentiated MSCs was checked by positive stainings with Alcian blue, toluidine, safranin O, and H&E. Chondrogenic ability was further confirmed by immunostaining for collagen type I, II, 2A1, aggrecan and collagen X.

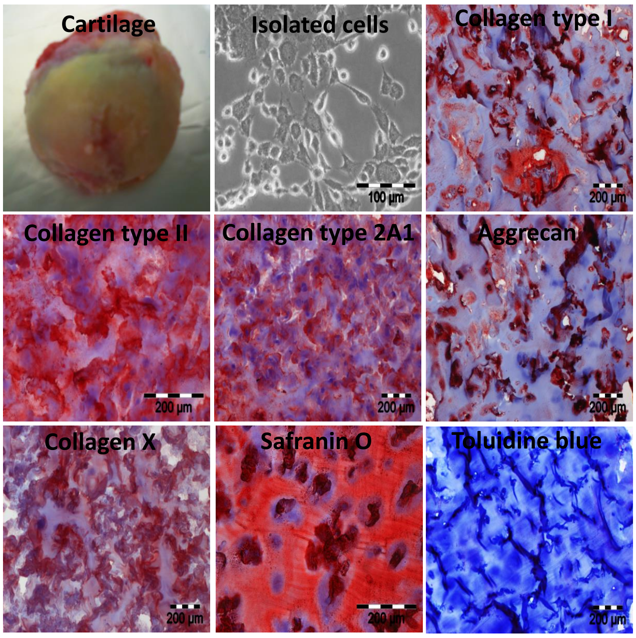

Figure S4: Chondrocytes isolated from native cartilage were stained for collagen type I, II, type 2A1, aggrecan, collagen X, Safranin O expressions and toluidine blue. These chondrocytes were used as the positive control for the experiments shown in Figure S3.

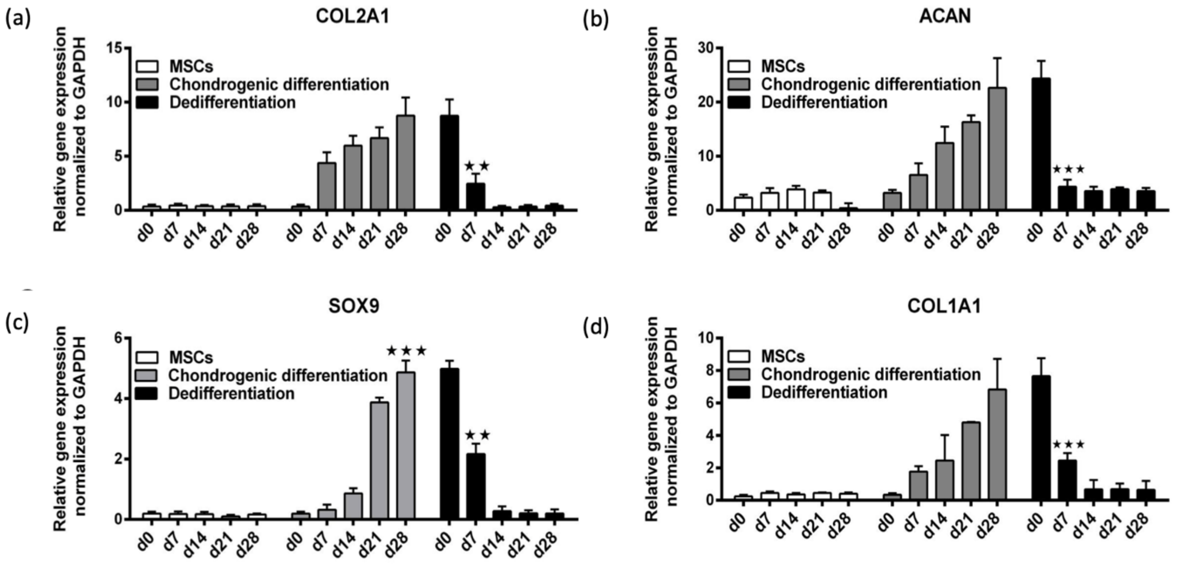

Figure S5: qPCR analysis of four genes that are of relevance for chondrogenic differentiation, namely (a) COL2A1, (b) ACAN, (c) SOX 9 and (d) COL1A1. The chondrogenic differentiation was confirmed by an upregulated expression of these genes when compared with negative controls and undifferentiated MSCs, day 0. Dedifferentiation was confirmed by a statistically significant downregulation of the four genes. Experiments were performed in triplicate. The statistical analysis was performed by using SigmaStat 3.5 software (Systat Software, USA), whereas GraphPad Prism4 (GraphPad Software) was used for drawing graphs. Simple Student´s t-test was used for statistical assessment, and asterisks were assigned in the order $P^* < 0.05$, $P^{**} < 0.01$, and $P^{***} < 0.001$ for statistically significant values, whereas exact *P* values were mentioned for statistically non-significant data sets. Error bars in all figures represent standard error of the mean.

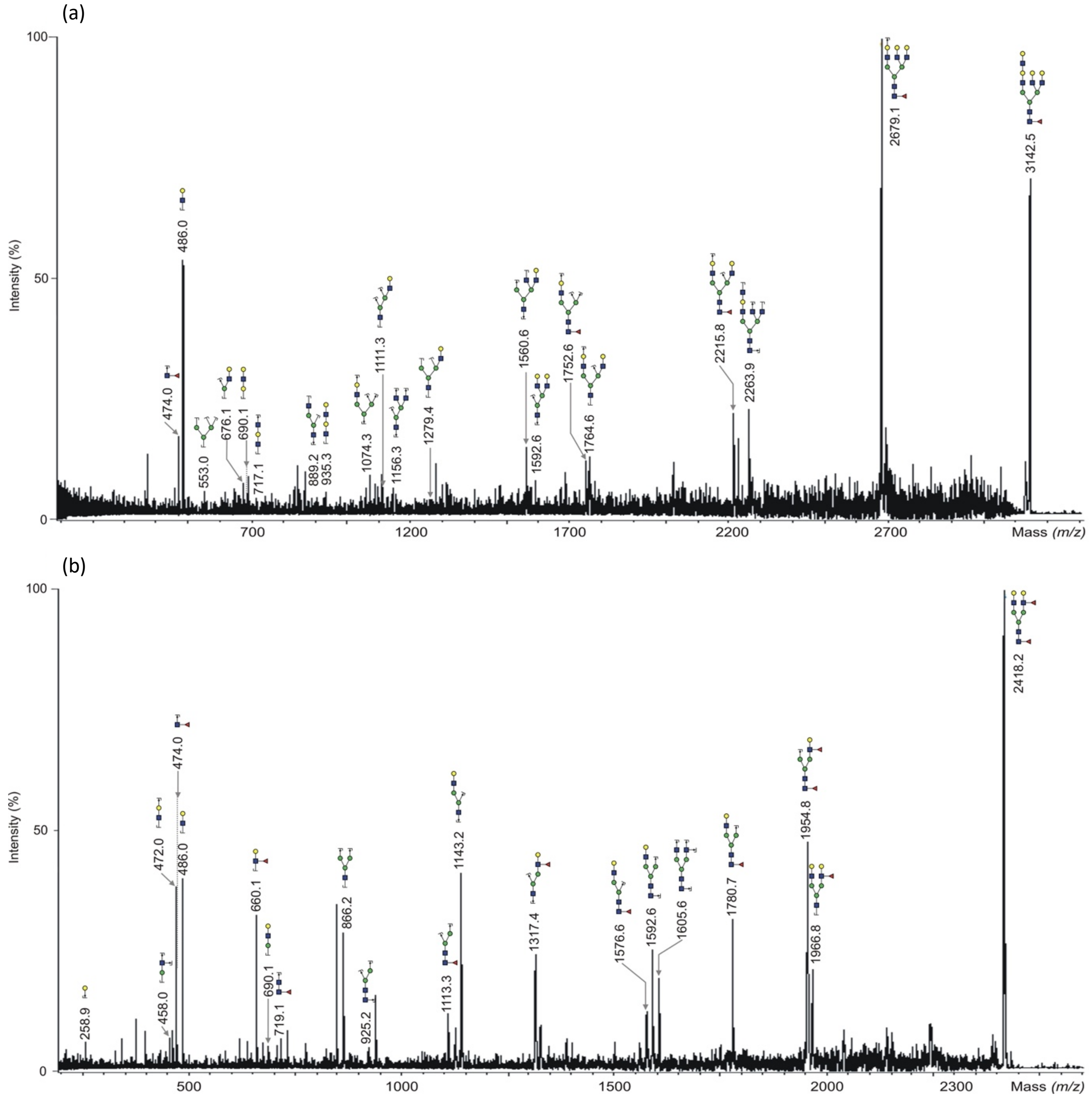

Figure S6. MALDI-TOF/TOF mass spectrum of *m/z* 3142.5 of the composition H7N6F1 derived from human MSCs **(a)** and of *m/z* 2418.2 of the composition H5N4F2 derived from day 28 chondrogenically differentiated MSCs **(b)**. Blue square represents *N*-acetylglucosamine, green circle mannose, yellow circle galactose, red triangle fucose and pink diamond *N*-acetylneuraminic acid.

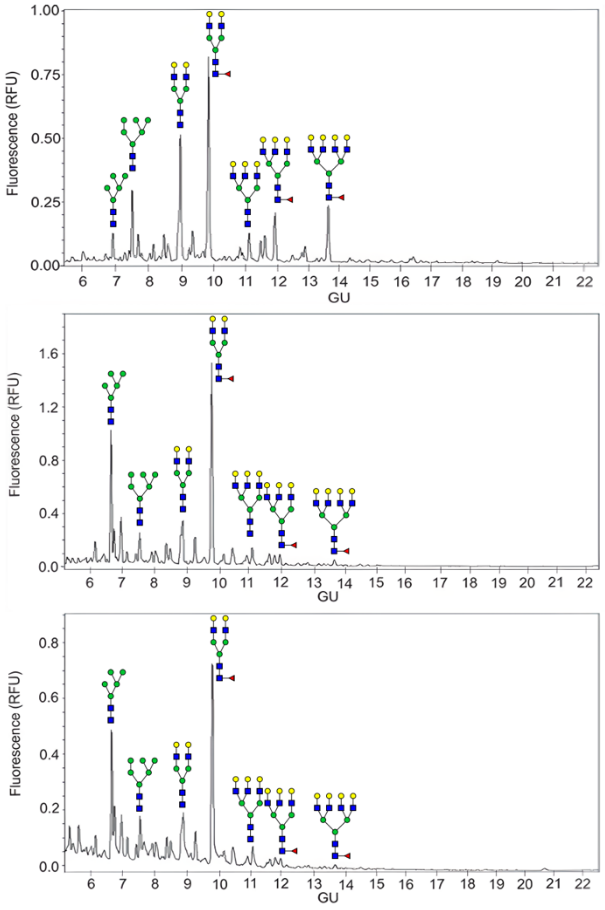

Figure S7. CE-LIF electropherograms of PNGase F-released and desialylated *N*-glycans from **(a)** undifferentiated, **(b)** day 5 and **(c)** day 28 chondrogenically differentiated human MSCs. Cells were digested with trypsin in order to release cell surface (glyco)peptides. (Glyco)peptides were subjected to PNGase F digestion to release *N*-glycans, desialylated and measured by CE-LIF. Blue square represents *N*-acetylglucosamine, green circle mannose, yellow circle galactose, red triangle fucose. Migration times are presented as glucose units because mobility varies from run to run as the electrolyte concentration changes [1, 2].

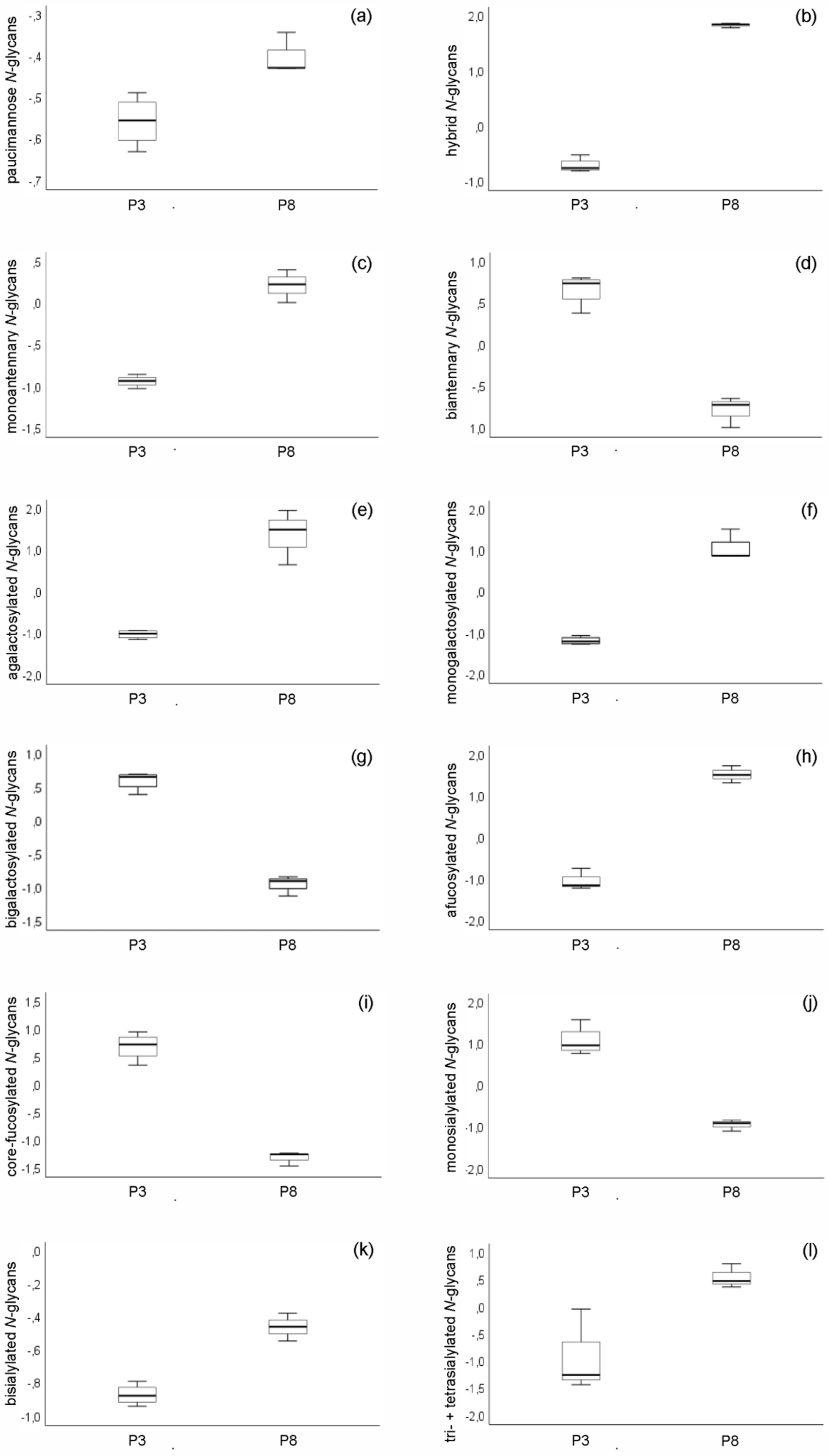
(a)
paucimannose N-glycans
-,3
-,4
-,5
-,6
-,7
P3
P8
(b)
hybrid N-glycans
2,0
1,0
,0
-1,0
P3
P8
(c)
monoantennary N-glycans
,5
,0
-,5
-1,0
-1,5
P3
P8
(d)
biantennary N-glycans
1,0
,5
,0
-,5
1,0
P3
P8
(e)
agalactosylated N-glycans
2,0
1,0
,0
-1,0
-2,0
P3
P8
(f)
monogalactosylated N-glycans
2,0
1,0
,0
-1,0
-2,0
P3
P8
(g)
bigalactosylated N-glycans
1,0
,5
,0
-,5
-1,0
-1,5
P3
P8
(h)
afucosylated N-glycans
2,0
1,0
,0
-1,0
-2,0
P3
P8
(i)
core-fucosylated N-glycans
1,5
1,0
,5
,0
-,5
-1,0
-1,5
P3
P8
(j)
monosialylated N-glycans
2,0
1,0
,0
-1,0
-2,0
P3
P8
(k)
bisialylated N-glycans
,0
-,2
-,4
-,6
-,8
-1,0
P3
P8
(l)
tri- + tetrasialylated N-glycans
1,0
,5
,0
-,5
-1,0
-1,5
-2,0
P3
P8

Figure S8. Box plots comparing the *N*-glycosylation traits of undifferentiated MSCs at passage 3 and passage 8. Given the low sample size within individual groups ($n < 5$), non-parametric correlation analyses were conducted using Spearman's rank correlation coefficient (ρ). To obtain robust significance estimates under small-sample conditions, exact *p*-values were calculated using a permutation approach with 5,000 iterations. Correlations were considered strong positive when $\rho \geq 0.8$, and strong negative when $\rho \leq -0.8$. P-value for all box plots were equal or lower to 0.057. Traits meeting this criterion included a) paucimannose *N*-glycans, (b) hybrid *N*-glycans, (c) monoantennary *N*-glycans, (d) biantennary *N*-glycans, (e) agalactosylated *N*-glycans, (f) monogalactosylated *N*-glycans, (g) bigalactosylated *N*-glycans, (h) afucosylated *N*-glycans, (i) core-fucosylated *N*-glycans, (j) monosialylated N-glycans, (k) bisialylated N-glycans, (l) tri + tetrasialylated N-glycans.

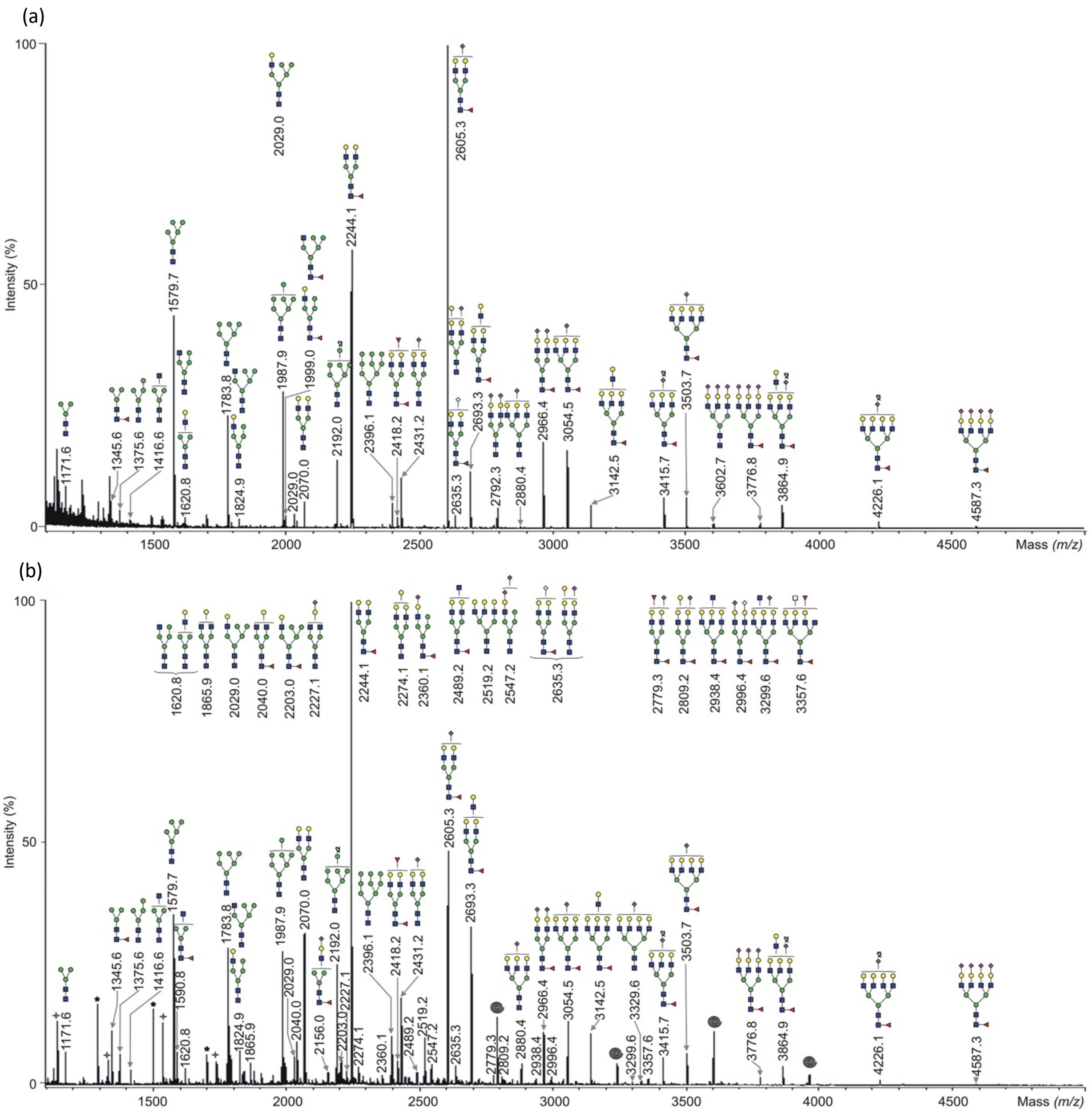

Figure S9. MALDI-TOF-MS of PNGase F-released *N*-glycans. A representative mass spectrum of **(a)** undifferentiated MSCs and **(b)** chondrogenic dedifferentiated MSCs is shown together with the 55 most abundant structures. Blue square represents *N*-acetylglucosamine, green circle mannose, yellow circle galactose, red triangle fucose and pink diamond *N*-acetylneuraminic acid. * Polyhexose contaminations. ✦Non identified peaks. ● Cell culture contaminations.

References

[1] Frisch, E., Schwedler, C., Kaup, M., Iona Braicu, E., *et al.*, Endo-beta-N-acetylglucosaminidase H de-N-glycosylation in a domestic microwave oven: application to biomarker discovery. *Anal Biochem* 2013, *433*, 65–69.
[2] Schwedler, C., Kaup, M., Weiz, S., Hoppe, M., *et al.*, Identification of 34 N-glycan isomers in human serum by capillary electrophoresis coupled with laser-induced fluorescence allows improving glycan biomarker discovery. *Anal Bioanal Chem* 2014, *406*, 7185–7193.
