# Supplementary material for: The N‐Glycome to Differentiate Mesenchymal Stem Cells Upon Chondrogenic Differentiation, Dedifferentiation, and Senescence
Source: Proteomics. 2026 Mar 25;26(7):17–27. doi: 10.1002/pmic.70124 (PMC13327702; doi:10.1002/pmic.70124)
Supplement: Supplementary file 3 — Supporting File 3: pmic70124‐sup‐0003‐Tables.pdf. [file PMIC-26--s001.pdf]

m/z	composition	% in MSC	% in day 5	% in day 28	*m/z*	composition	% in MSC	% in day 5	% in C day 28
1171.6	H3N2	0.32	0.07	0.60	2396.1	H9N2	0.82	0.30	1.02
1345.6	H3N2F1	0.27	0.17	5.04	2401.1	S1H4N4F1	0.14	0.12	0.28
1375.6	H4N2	0.21	0.07	0.39	2418.2	H5N4F2	0.49	0.91	0.73
1416.7	H3N3	0.11	0.03	0.07	2431.2	S1H5N4	2.17	3.15	2.58
1549.7	H4N2F1	0.00	0.00	0.08	2448.2	H6N4F1	0.02	0.03	0.02
1579.7	H5N2	4.84	2.64	5.23	2461.2	G1H5N4	0.09	0.05	0.06
1590.8	H3N3F1	0.12	0.20	0.19	2489.2	H5N5F1	0.07	0.02	0.02
1620.8	H4N3	0.06	0.06	0.07	2547.2	S2H5N3	0.07	0.02	0.06
1753.8	H5N2F1	0.00	0.00	0.04	2564.2	S1H6N3F1	0.07	0.01	0.02
1783.8	H6N2	3.21	1.14	2.35	2592.3	H5N4F3	0.01	0.00	0.00
1794.9	H4N3F1	0.15	0.22	0.29	2599.3	H10N2	0.00	0.02	0.00
1824.9	H5N3	0.22	0.22	0.17	2605.3	S1H5N4F1	38.00	40.81	30.83
1835.9	H3N4F1	0.05	0.02	0.04	2635.3	S1H6N4	0.57	0.28	0.29
1865.9	H4N4	0.07	0.11	0.10	2646.3	S1H4N5F1	0.00	0.05	0.02
1968.9	H4N3F2	0.00	0.00	0.07	2693.3	H6N5F1	2.98	1.09	0.41
1981.9	S1H4N3	0.07	0.03	0.20	2779.3	S1H5N4F2	0.17	0.48	1.90

1987.9	H7N2	5.57	0.86	1.52	2809.3	S1H6N4F1	0.06	0.03	0.03
1999.0	H5N3F1	0.34	0.15	0.08	2822.4	S1G1H5N4	0.01	0.01	0.02
2029.0	H6N3	0.48	0.22	0.21	2850.4	S1H5N5F1	0.06	0.01	0.02
2040.0	H4N4F1	0.33	0.43	0.46	2880.4	S1H6N5	0.14	0.03	0.03
2070.0	H5N4	0.97	2.35	1.23	2966.4	S2H5N4F1	4.60	7.81	15.85
2081.0	H3N5F1	0.00	0.10	0.79	2979.4	H5N7F1	0.00	0.00	0.02
2156.0	S1H4N3F1	0.10	0.12	3.39	2996.4	S2H6N4	0.09	0.03	0.05
2186.0	S1H5N3	0.20	0.15	0.32	3026.4	G2H5N4F1	0.02	0.01	0.01
2192.0	H8N2	2.38	0.73	1.36	3054.5	S1H6N5F1	4.22	1.80	1.30
2203.0	H6N3F1	0.18	0.03	0.03	3142.5	H7N6F1	1.22	0.07	0.03
2214.1	H4N4F2	0.04	0.01	0.03	3228.6	S1H6N5F2	0.00	0.02	0.05
2227.1	S1H4N4	0.04	0.04	0.05	3402.6	S1H6N5F3	0.00	0.00	0.01
2244.1	H5N4F1	16.74	29.46	13.31	3415.7	S2H6N5F1	1.50	1.33	2.31
2274.1	H6N4	0.03	0.05	0.03	3503.7	S1H7N6F1	1.52	0.09	0.06
2285.1	H4N5F1	0.02	0.08	0.05	3776.8	S3H6N5F1	0.13	0.41	1.07
2326.1	H3N6F1	0.00	0.03	0.02	3864.9	S2H7N6F1	1.09	0.11	0.08
2360.1	S1H5N3F1	0.22	0.08	0.12	4226.1	S3H7N6F1	0.46	0.02	0.08
2390.1	S1H6N3	0.15	0.07	0.24	4587.3	S4H7N6F1	0.09	0.01	0.01

Supplementary Table S1. Average relative amounts of all PNGase F-released *N*-glycans of undifferentiated and chondrogenically differentiated human MSCs on day 5 and day 28 of differentiation derived from three different donors. The extracellular matrix was removed prior to the isolation of differentiated MSCs. H: hexose, N: N-acetylhexosamine, F: deoxyhexose, S: N-acetylneuraminic acid, G: N-glycolylneuraminic acid.

Found N-glycan structures			Cell type		Consecutive Enzymatic Digestions					
m/z	Composition	Proposed Structure	MSC	C day 28	N	G4	F3,4	G4	F	M
1171.6	H3N2	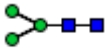	x	x	ud	ud	ud	ud	ud	d
1345.6	H3N2F1	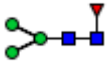	x	x	ud	ud	ud	ud	d	d
1375.6	H4N2	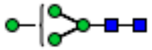	x	x	ud	ud	ud	ud	ud	d
1416.7	H3N3	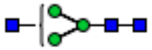	x	x	ud	ud	ud	ud	ud	d
1579.7	H5N2	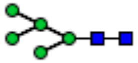	x	x	ud	ud	ud	ud	ud	d
1590.8	H3N3F1	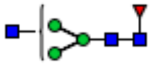	x	x	ud	ud	ud	ud	d	d
1620.8	H4N3	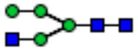	x	x	ud	ud	ud	ud	ud	ud
		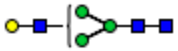								
1783.8	H6N2	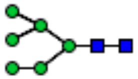	x	x	ud	ud	ud	ud	ud	d
1794.9	H4N3F1	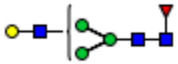	x	x	ud	ud	ud	ud	d	d
		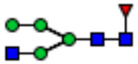								

1824.9	H5N3	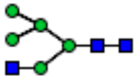	x	x	ud	ud	ud	ud	ud	d
		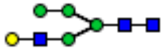								
1865.9	H4N4	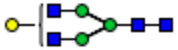	x	x	ud	ud	ud	d	ud	ud
1987.9	H7N2	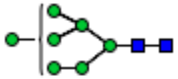	x	x	ud	ud	ud	ud	ud	d
1999.0	H5N3F1	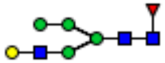	x	x	ud	d	ud	ud	d	d
2029.0	H6N3	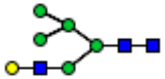	x	x	ud	d1	ud	ud	ud	d
2040.0	H4N4F1	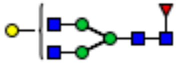	x	x	ud	ud	ud	ud	d	ud
2070.0	H5N4	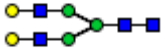	x	x	ud	d	ud	ud	ud	ud
2081.0	H3N5F1	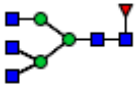		x	ud	ud	ud	ud	d	ud
		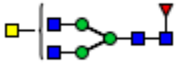								
2192.0	H8N2	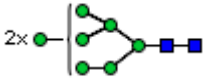	x	x	ud	ud	ud	ud	ud	d
2203.0	H6N3F1	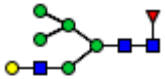	x	x	ud	d	ud	ud	d	d

2244.1	H5N4F1	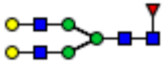	x	x	ud	d	ud	ud	d	ud
2360.1	S1H5N3F1	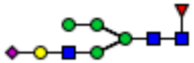	x	x	d	d	ud	ud	d	d
2390.1	S1H6N3	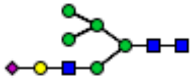	x	x	d	d	ud	ud	ud	d
2396.1	H9N2	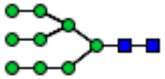	x	x	ud	ud	ud	ud	ud	d
2401.1	S1H4N4F1	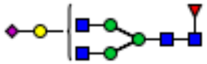	x	x	d	ud	ud	ud	d	ud
2418.2	H5N4F2	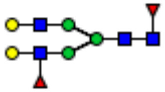	x	x	ud	d	d	d	d	ud
2431.2	S1H5N4	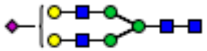	x	x	d	d	ud	ud	ud	ud
2461.2	G1H5N4	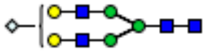	x	x	d	d	ud	ud	ud	ud
2489.2	H5N5F1	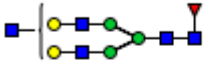	x	x	ud	d	ud	ud	d1	ud
2605.3	S1H5N4F1	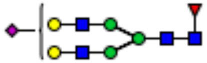	x	x	d	d	ud	ud	d	ud
2693.3	H6N5F1	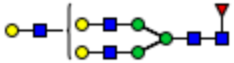	x	x	ud	d	ud	ud	d	ud
2779.3	S1H5N4F2	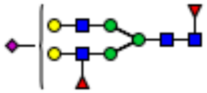	x	x	d	d	d	d	d	ud

2792.3	S2H5N4	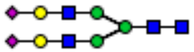	x	x	d	d	ud	ud	ud	ud
2822.4	S1G1H5N4	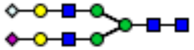	x	x	d	d	ud	ud	ud	ud
2850.4	S1H5N5F1	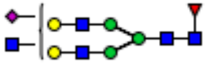	x	x	d	d	ud	ud	d	ud
2880.4	S1H6N5	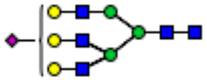	x	x	d	d	ud	ud	ud	ud
2966.4	S2H5N4F1	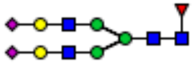	x	x	d	d	ud	ud	d	ud
2996.4	S1G1H5N4F1	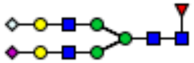	x	x	d	d	ud	ud	d	ud
3026.4	G2H5N4F1	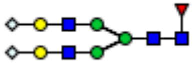	x	x	d	d	ud	ud	d	ud
3054.5	S1H6N5F1	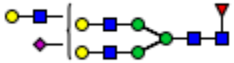	x	x	d	d	ud	ud	d	ud
3142.5	H7N6F1	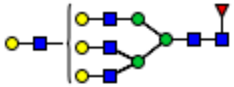	x	x	ud	d	ud	ud	d	ud
3228.6	S1H6N5F2	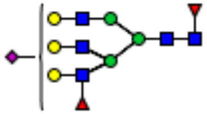		x	d	d	d	d	d	ud
3241.6	S2H6N5	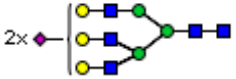	x	x	d	d	ud	ud	ud	ud
3415.7	S2H6N5F1	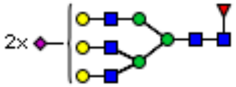	x	x	d	d	ud	ud	d	ud

3503.7	S1H7N6F1	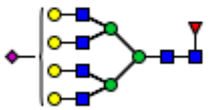	x	x	d	d	ud	ud	d	ud
3602.7	S3H6N5	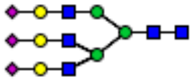	x	x	d	d	ud	ud	ud	ud
3776.8	S3H6N5F1	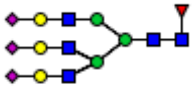	x	x	d	d	ud	ud	d	ud
3864.9	S2H7N6F1	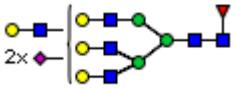	x	x	d	d	ud	ud	d	ud
4226.1	S3H7N6F1	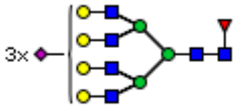	x	x	d	d	ud	ud	d	ud
4587.3	S4H7N6F1	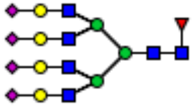	x	x	d	d	ud	ud	d	ud

Supplementary Table S2. Exoglycosidase digestions of PNGase F-released N-glycans derived from undifferentiated MSCs and day 28 of chondrogenically differentiated MSCs. N: *Arthrobacter ureafaciens* neuraminidase, G4: β(1–4) galactosidase from *Streptococcus pneumoniae*, F3,4: almond meal α(1–3,4) fucosidase , F: Bovine kidney α(1-2,3,4,6) fucosidase, M: αMannosidase from *Canavalia ensiformis*. The presence of Lewis^{X} antenna was established by exoglycosidase digestions. The cartoons imply neither a specific glycan isomer nor allocation of the Lewis^{X} motif to a distinct antennae. Note that some low abundant structures were not observed in the mass spectrum after exoglycosidase digestion and were therefore not listed here. d: digested, ud: undigested, H: hexose, N: N-acetylhexosamine, F: deoxyhexose, S: N-acetylneuraminic acid, G: N-glycolylneuraminic acid. Blue square represents N-acetylglucosamine, green circle mannose, yellow circle galactose, red triangle fucose, pink diamond N-acetylneuraminic acid and white diamond N-glycolylneuraminic acid.

m/z	composition	Diagnostic fragments
1590.8	H3N3F1	Core fucose: 474.0 (N1F1), 719.1 (N2F1)
1999.0	H5N3F1	Core fucose: 474.0 (N1F1), 719.1 (N2F1)
2040.0	H4N4F1	Core fucose: 474.0 (N1F1), 719.1 (N2F1)
2244.1	H5N4F1	Core fucose: 474.0 (N1F1), 719.1 (N2F1)
2360.1	S1H5N3F1	Core fucose: 719.1 (N2F1), sialylated antenna: 847.2 (H1N1S1)
2418.2	H5N4F2	Lewis ^{x} antenna: 660.1 (H1N1F1), core fucose: 474.0 (N1F1), 719.1 (N2F1)
2431.2	S1H5N4	sialylated antenna: 847.2 (H1N1S1)
2605.3	S1H5N4F1	core fucose: 719.1 (N2F1), sialylated antenna: 847.2 (H1N1S1)
2693.3	H6N5F1	core fucose: 719.1 (N2F1)
2779.3	S1H5N4F2	core fucose: 474.0 (N1F1), sialylated antenna: 847.2 (H1N1S1)
2792.3	S2H5N4	sialylated antenna: 847.2 (H1N1S1)
2966.4	S2H5N4F1	core fucose: 719.1 (N2F1). sialylated antenna: 847.2 (H1N1S1)
3054.5	S1H6N5F1	core fucose: 474.0 (N1F1), 719.1 (N2F1)
3142.5	H7N6F1	LacNAc motif: 935.3 (H2N2) in MSC undiff

Supplementary Table S3. List of *N*-glycans signals there were fragmented by MALDI-TOF/TOF-MS, diagnostic fragments are indicated in the Table.

		type				glycosylation traits															
m/*z*	Composition	Paucimannosylation	high-mannoses	hybrid	complex	mono ant	bi ant	tri ant	tetra ant	0 Gal	1 Gal	2 Gal	3 Gal	4 Gal	0 Fuc	1 Fuc	2 +3 Fuc	0 SA	1 SA	2 SA	3+4 SA
1171.6	H3N2		X																		
1345.6	H3N2F1	X																			
1375.6	H4N2		X																		
1416.7	H3N3				X	X				X					X			X			
1549.7	H4N2F1	X																			
1579.7	H5N2		X																		
1590.8	H3N3F1				X	X				X						X		X			
1620.8	H4N3			X																	
1753.8	H5N2F1	X																			
1783.8	H6N2		X																		
1794.9	H4N3F1				X	X					X					X		X			
1824.9	H5N3			X																	
1835.9	H3N4F1				X		X			X						X		X			

1865.9	H4N4			X		X			X		X			X	
1968.9	H4N3F2			X	X				X				X	X	
1981.9	S1H4N3			X	X				X		X				X
1987.9	H7N2	X													
1999.0	H5N3F1		X												
2029.0	H6N3		X												
2040.0	H4N4F1			X		X			X			X		X	
2070.0	H5N4			X		X				X	X			X	
2081.0	H3N5F1			X			X	X				X		X	
2156.0	S1H4N3F1			X	X				X			X			X
2186.0	S1H5N3		X												
2192.0	H8N2	X													
2203.0	H6N3F1		X												
2214.1	H4N4F2			X		X			X				X	X	
2227.1	S1H4N4			X		X			X		X				X
2244.1	H5N4F1			X		X				X		X		X	

2274.1	H6N4		X												
2285.1	H4N5F1			X		X			X			X		X	
2326.1	H3N6F1			X			X	X				X		X	
2360.1	S1H5N3F1		X												
2390.1	S1H6N3		X												
2396.1	H9N2	X													
2401.1	S1H4N4F1			X	X				X			X			X
2418.2	H5N4F2			X	X					X			X	X	
2431.2	S1H5N4			X	X					X	X				X
2448.2	H6N4F1		X												
2461.2	G1H5N4			X	X					X	X				X
2489.2	H5N5F1			X		X				X		X		X	
2547.2	S2H5N3		X												
2564.2	S1H6N3F1		X												

2592.3	H5N4F3			X	X				X				X	X		
2599.3	H10N2	X														
2605.3	S1H5N4 F1			X	X				X			X			X	
2635.3	S1H6N4		X													
2646.3	S1H4N5 F1			X		X		X				X			X	
2693.3	H6N5F1			X		X				X		X		X		
2779.3	S1H5N4 F2			X	X				X				X		X	
2809.3	S1H6N4 F1		X													
2822.4	S1G1H5 N4			X	X				X		X					X
2850.4	S1H5N5 F1			X		X			X			X			X	
2880.4	S1H6N5			X		X				X	X				X	
2966.4	S2H5N4 F1			X	X				X			X				X
2979.4	H5N7F1			X			X		X			X		X		

2996.4	S2H6N4	X													
3026.4	G2H5N4F1		X	X			X			X				X	
3054.5	S1H6N5F1		X		X			X		X			X		
3142.5	H7N6F1		X			X			X	X		X			
3228.6	S1H6N5F2		X		X			X			X		X		
3357.6	H6N7F2		X			X		X			X	X			
3402.6	S1H6N5F3		X		X			X			X		X		
3415.7	S2H6N5F1		X		X			X		X				X	
3503.7	S1H7N6F1		X			X			X	X			X		
3776.8	S3H6N5F1		X		X			X		X					X
3864.9	S2H7N6F1		X			X			X	X				X	

4226.1	S3H7N6 F1	X	X	X	X	X
4587.3	S4H7N6 F1	X	X	X	X	X

Supplementary Table S4. The assigned N-glycans were grouped according to glycan structural features: type (paucimannose, high-mannose, hybrid, complex) and glycosylation traits (antennarity, galactosylation, fucosylation and sialylation).

descriptive statistics

**trait**	**MSC cells**	**n. of cases**	**mean**	**SE**	**median**	**SD**	**range**	**min**	**max**
pauci-manose	undiff	4	1,089	0,021	1,087	0,044	0,079	1,051	1,129
	day 5	3	0,177	0,085	0,130	0,147	0,282	0,060	0,342
	day 28	3	5,270	1,255	5,596	2,174	4,311	2,951	7,262
	P8	3	0,549	0,059	0,492	0,102	0,177	0,490	0,667
	dediff	4	0,227	0,062	0,232	0,124	0,292	0,077	0,369
high-mannose	undiff	4	31,431	6,834	31,677	13,667	28,377	16,997	45,374
	day 5	3	5,926	1,767	6,726	3,061	5,963	2,545	8,508
	day 28	3	12,769	3,268	10,699	5,661	10,739	8,434	19,173
	P8	3	26,565	2,181	25,798	3,778	7,439	23,230	30,669
	dediff	4	17,799	3,003	18,176	6,007	14,645	10,099	24,744
hybrid	undiff	4	7,702	0,201	7,767	0,403	0,840	7,216	8,056
	day 5	3	1,449	0,322	1,749	0,557	0,986	0,806	1,792
	day 28	3	1,758	0,247	1,511	0,428	0,741	1,511	2,253

**trait**	**MSC cells**	**n. of cases**	**mean**	**SE**	**median**	**SD**	**range**	**min**	**max**
hybrid	P8	3	15,275	0,129	15,358	0,224	0,423	15,022	15,445
	dediff	4	1,614	0,350	1,367	0,700	1,554	1,083	2,637
monoant	undiff	4	1,836	0,166	1,705	0,332	0,713	1,611	2,324
	day 5	3	0,627	0,157	0,516	0,272	0,509	0,4285	0,937
	day 28	3	4,301	0,808	4,762	1,399	2,682	2,729	5,411
	P8	3	2,061	0,172	2,084	0,297	0,593	1,753	2,346
	dediff	4	0,338	0,051	0,344	0,102	0,249	0,2079	0,457
biant	undiff	4	39,622	4,206	39,568	8,413	16,431	31,461	47,892
	day 5	3	89,371	2,505	88,398	4,338	8,511	85,602	94,113
	day 28	3	69,326	3,848	68,437	6,665	13,241	63,150	76,391
	P8	3	48,517	2,088	49,809	3,617	6,879	44,431	51,310
	dediff	4	77,138	1,906	78,602	3,811	8,342	71,504	79,845
triant	undiff	4	12,343	1,867	12,454	3,733	8,994	7,734	16,728
	day 5	3	2,267	0,276	2,404	0,478	0,926	1,735	2,661
	day 28	3	6,288	1,519	7,649	2,630	4,702	3,256	7,958

trait	MSC cells	n. of cases	mean	SE	median	SD	range	min	max
triant	P8	3	5,015	0,196	5,149	0,340	0,639	4,628	5,267
	dediff	4	2,041	1,037	1,147	2,073	4,393	0,740	5,133
tetraant	undiff	4	5,978	1,282	6,043	2,564	6,240	2,793	9,033
	day 5	3	0,183	0,022	0,165	0,037	0,068	0,158	0,226
	day 28	3	0,289	0,074	0,335	0,128	0,242	0,145	0,387
	P8	3	2,018	0,075	1,978	0,130	0,251	1,912	2,163
	dediff	4	0,842	0,347	0,579	0,693	1,527	0,341	1,868
agal	undiff	4	1,629	0,178	1,519	0,357	0,799	1,339	2,138
	day 5	3	0,406	0,153	0,289	0,264	0,488	0,221	0,709
	day 28	3	1,128	0,459	0,726	0,795	1,431	0,613	2,044
	P8	3	2,352	0,346	2,469	0,599	1,180	1,704	2,884
	dediff	4	0,185	0,046	0,200	0,092	0,189	0,076	0,265
monogal	undiff	4	3,454	0,366	3,246	0,731	1,584	2,870	4,454
	day 5	3	1,237	0,223	1,060	0,386	0,708	0,971	1,679
	day 28	3	5,079	0,723	5,323	1,252	2,468	3,724	6,192
	P8	3	5,266	0,450	4,823	0,780	1,358	4,808	6,166

trait	MSC cells	n. of cases	mean	SE	median	SD	range	min	max
monogal	dediff	4	0,521	0,100	0,479	0,201	0,441	0,344	0,785
bigal	undiff	4	37,715	4,022	37,586	8,043	15,962	29,863	45,825
	day 5	3	86,155	3,534	84,902	6,121	12,047	80,757	92,805
	day 28	3	68,393	3,757	67,566	6,508	12,936	62,338	75,274
	P8	3	41,574	1,704	42,594	2,951	5,631	38,248	43,880
	dediff	4	72,331	1,416	73,462	2,833	6,089	68,156	74,245
trigal	undiff	4	11,588	1,868	11,667	3,736	8,978	7,020	15,999
	day 5	3	4,328	1,131	4,860	1,959	3,809	2,158	5,967
	day 28	3	5,344	1,322	6,028	2,290	4,424	2,790	7,214
	P8	3	4,933	0,418	4,756	0,724	1,414	4,313	5,728
	dediff	4	5,663	1,429	4,638	2,858	6,219	3,579	9,798
tetragal	undiff	4	5,393	1,241	5,368	2,481	6,077	2,380	8,457
	day 5	3	0,322	0,038	0,352	0,066	0,121	0,246	0,367
	day 28	3	0,259	0,066	0,279	0,115	0,227	0,136	0,363
	P8	3	3,485	0,205	3,452	0,354	0,707	3,149	3,855

trait	MSC cells	n. of cases	mean	SE	median	SD	range	min	max
tetragal	dediff	4	1,659	0,601	1,189	1,220	2,658	0,800	3,457
gal_total	undiff	4	14,538	1,715	14,531	3,431	7,046	11,021	18,067
	day 5	3	23,010	0,563	22,794	0,975	1,913	22,162	24,075
	day 28	3	19,769	1,156	20,037	2,002	3,976	17,647	21,623
	P8	3	13,814	0,557	14,246	0,965	1,779	12,709	14,488
	dediff	4	20,044	0,712	20,062	1,425	3,472	18,289	21,761
afuc	undiff	4	10,173	0,661	10,208	1,322	2,835	8,721	11,556
	day 5	3	5,876	1,723	6,117	2,985	5,954	2,779	8,733
	day 28	3	4,441	0,812	4,012	1,406	2,712	3,299	6,011
	P8	3	14,276	0,548	14,231	0,949	1,897	13,350	15,247
	dediff	4	2,465	0,488	2,083	0,976	2,131	1,782	3,914
monofuc	undiff	4	47,781	6,056	48,233	12,112	25,529	34,565	60,094
	day 5	3	85,126	3,847	81,325	6,663	11,586	81,234	92,820
	day 28	3	72,898	5,017	76,280	8,690	16,362	63,026	79,388
	P8	3	38,433	1,443	39,696	2,499	4,495	35,555	40,050
	dediff	4	77,604	2,434	78,293	4,869	11,667	71,081	82,748

trait	MSC cells	n. of cases	mean	SE	median	SD	range	min	max
bifuc	undiff	4	1,824	0,150	1,775	0,300	0,695	1,527	2,221
	day 5	3	1,445	0,299	1,337	0,517	1,017	0,991	2,008
	day 28	3	2,854	0,427	2,601	0,739	1,412	2,276	3,687
	P8	3	4,156	0,261	4,039	0,453	0,882	3,773	4,655
	dediff	4	0,278	0,067	0,230	0,135	0,298	0,178	0,475
trifuc	undiff	4	0,000	0,000	0,000	0,000	0,000	0,000	0,000
	day 5	3	0,000	0,000	0,000	0,000	0,000	0,000	0,000
	day 28	3	0,010	0,005	0,013	0,009	0,016	0,000	0,017
	P8	3	0,745	0,074	0,724	0,130	0,255	0,629	0,884
	dediff	4	0,012	0,012	0,000	0,025	0,050	0,000	0,050
fuc_total	undiff	4	16,535	2,066	16,670	4,131	8,741	12,031	20,772
	day 5	3	28,857	1,208	27,747	2,092	3,716	27,554	31,270
	day 28	3	25,254	1,790	26,298	3,100	5,930	21,767	27,697
	P8	3	14,445	0,573	14,819	0,993	1,877	13,319	15,196
	dediff	4	25,965	0,827	26,163	1,654	3,981	23,776	27,758
asial	undiff	4	33,001	4,844	32,551	9,688	18,349	24,277	42,626

trait	MSC cells	n. of cases	mean	SE	median	SD	range	min	max
	day 5	3	35,608	1,131	34,562	1,959	3,473	34,395	37,868
	day 28	3	18,352	3,023	19,008	5,236	10,410	12,818	23,228
asial	P8	3	27,754	0,941	28,340	1,630	3,098	25,912	29,010
	dediff	4	25,394	0,950	25,522	1,899	4,136	23,199	27,335
mono-sial	undiff	4	20,153	1,956	19,720	3,912	9,134	16,019	25,152
	day 5	3	46,982	3,430	44,004	5,941	10,705	43,118	53,823
	day 28	3	41,899	1,212	42,506	2,099	4,064	39,563	43,628
	P8	3	23,495	1,108	24,022	1,919	3,729	21,367	25,096
	dediff	4	51,876	2,476	50,406	4,951	11,320	47,687	59,007
bisial	undiff	4	5,381	0,817	5,325	1,635	3,420	3,727	7,147
	day 5	3	9,388	0,810	9,866	1,403	2,680	7,808	10,489
	day 28	3	18,755	0,375	18,421	0,650	1,165	18,339	19,504
	P8	3	5,174	0,277	5,182	0,480	0,960	4,690	5,649
	dediff	4	2,826	0,179	2,793	0,359	0,860	2,430	3,290
tri + tetra_sial	undiff	4	1,244	0,334	1,122	0,668	1,383	0,675	2,057
	day 5	3	0,470	0,084	0,543	0,145	0,260	0,304	0,564

trait	MSC cells	n. of cases	mean	SE	median	SD	range	min	max
	day 28	3	1,198	0,357	1,175	0,618	1,235	0,592	1,827
	P8	3	1,188	0,077	1,145	0,134	0,257	1,080	1,338
	dediff	4	0,263	0,193	0,108	0,386	0,837	0,000	0,837
sial_total	undiff	4	8,926	0,913	8,804	1,827	4,453	6,822	11,274
	day 5	3	18,947	0,901	18,266	1,560	2,889	17,842	20,732
	day 28	3	20,617	0,564	21,062	0,976	1,794	19,498	21,292
	P8	3	9,952	0,434	10,116	0,752	1,477	9,132	10,608
	dediff	4	18,322	0,908	17,821	1,815	4,164	16,741	20,905

Supplementary Table S5. Descriptive statistics were used to summarize the data and are presented as appropriate measures of central tendency and variability for all N-glycosylation traits (Supplementary Material Table S4). SE, standard error; SD, standard deviation.
